# Urban landscape and street-design factors associated with road-traffic mortality in Latin America between 2010 and 2016 (SALURBAL): an ecological study

**DOI:** 10.1016/S2542-5196(21)00323-5

**Published:** 2022-02-09

**Authors:** D Alex Quistberg, Philipp Hessel, Daniel A Rodriguez, Olga L Sarmiento, Usama Bilal, Waleska Teixeira Caiaffa, J Jaime Miranda, Maria de Fatima de Pina, Akram Hernández-Vásquez, Ana V Diez Roux

**Affiliations:** a Urban Health Collaborative, Dornsife School of Public Health, Drexel University, Philadelphia, PA, USA; b Department of Environmental and Occupational Health, Dornsife School of Public Health, Drexel University, Philadelphia, PA, USA; c Department of Epidemiology & Biostatistics, Dornsife School of Public Health, Drexel University, Philadelphia, PA, USA; d Escuela de Gobierno Alberto Lleras Camargo, Universidad de Los Andes, Bogotá, Colombia; e City + Regional Planning and Institute for Transportation Studies, University of California, Berkeley, CA, USA; f School of Medicine, Universidad de los Andes, Bogotá, Colombia; g Observatório de Saúde Urbana em Belo Horizonte, Universidade Federal de Minas Gerais, Belo Horizonte, Brazil; h CRONICAS Center of Excellence in Chronic Diseases, Universidad Peruana Cayetano Heredia, Lima, Peru; i School of Medicine, Universidad Peruana Cayetano Heredia, Lima, Peru; j Instituto de Comunicação e Investigação Científica e Tecnológica em Saúde, Fundação Oswaldo Cruz, Rio de Janeiro, Brazil; k i3S—Instituto de Investigação e Inovação em Saúde, Universidade do Porto, Porto, Portugal

## Abstract

**Background:**

Road-traffic injuries are a key cause of death and disability in low-income and middle-income countries, but the effect of city characteristics on road-traffic mortality is unknown in these countries. The aim of this study was to determine associations between city-level built environment factors and road-traffic mortality in large Latin American cities.

**Methods:**

We selected cities from Argentina, Brazil, Chile, Colombia, Costa Rica, El Salvador, Guatemala, Mexico, Panama, and Peru; cities included in the analysis had a population of at least 100 000 people. We extracted data for road-traffic deaths that occurred between 2010 and 2016 from country vital registries. Deaths were grouped by 5-year age groups and sex. Road-traffic deaths were identified using ICD-10 codes, with adjustments for ill-defined codes and incomplete registration. City-level measures included population, urban development, street design, public transportation, and social environment. Associations were estimated using multilevel negative binomial models with robust variances.

**Findings:**

366 cities were included in the analysis. There were 328 408 road-traffic deaths in nearly 3·5 billion person-years across all countries, with an average crude rate of 17·1 deaths per 100 000 person-years. Nearly half of the people who died were younger than 35 years. In multivariable models, road-traffic mortality was higher in cities where urban development was more isolated (rate ratio [RR] 1·05 per 1 SD increase, 95% CI 1·02–1·09), but lower in cities with higher population density (0·94, 0·90–0·98), higher gross domestic product per capita (0·96, 0·94–0·98), and higher intersection density (0·92, 0·89–0·95). Cities with mass transit had lower road mortality rates than did those without (0·92, 0·86–0·99).

**Interpretation:**

Urban development policies that reduce isolated and disconnected urban development and that promote walkable street networks and public transport could be important strategies to reduce road-traffic deaths in Latin America and elsewhere.

**Funding:**

Wellcome Trust.

## Introduction

Urbanisation is increasing globally, with increased motorisation and urban sprawl being linked to road-traffic injuries and deaths. In Latin America—one of the most urbanised regions of the world with more than 80% of the population living in cities—road-traffic injuries are a leading cause of death for all ages;^1^, ^2^, ^3^ regionally, they are the leading cause of death among children aged 5–14 years, and they are the second leading cause of death among people aged 15–44 years.^4^ The economic effect of road injuries in the region is estimated to be a loss of 4·4% of annual gross domestic product (GDP).^1^ Road-traffic death rates vary across Latin American countries, but the overall regional rate has remained close to 20 deaths per 100 000 population annually since 1980, whereas the rate over that same period has halved in high-income countries.^1^

Cities can be designed and managed to improve road safety by reducing unsafe travel behaviours (eg, travelling at high speed), protecting vulnerable road users (eg, pedestrians), reducing volume of motor vehicle traffic, and prioritising active and public transportation.^5^, ^6^, ^7^ Existing evidence, mostly from studies in high-income settings, suggests road safety can be improved by intervening at the street level (eg, narrower roads, requiring motorists to stop for pedestrians),^5^, ^8^, ^9^ neighbourhood level (eg, increasing street connectivity and walkability, traffic calming),^5^, ^8^, ^9^ and potentially the city level (eg, increasing population density, reducing peripheral growth, increasing availability of public transportation).^6^, ^10^, ^11^

A few studies in Latin America have examined the effect of city or sub-city built environment features on road-traffic injuries and deaths,^12^, ^13^, ^14^, ^15^ but the focus has been on a single city or neighbourhood. No studies, of which we are aware, have examined city-level built environment characteristics in relation to road mortality using city-level vital registry data across a large heterogenous sample of cities in the region. Identifying which built environment factors in Latin American cities are associated with road-traffic mortality is key to achieving Sustainable Development Goal 3.6 of halving road-traffic deaths because urban design can play a fundamental role in promoting long-term road safety.^9^, ^16^

**Research in context**

**Evidence before this study**

We searched PubMed, ISI Web of Knowledge, SciELO, and Transportation Research International Documentation for articles published from inception up to Jan 31, 2020, examining road-traffic deaths in Latin America in relation to the built environment at the city level using the following search terms in English, Spanish, and Portuguese: (Latin America) AND (built environment) AND (“road mortality” OR “road traffic mortality” OR “traffic mortality” OR “road injury” OR “road traffic injury” OR “traffic injury” OR “road accidents” OR “motor vehicle collisions” OR “pedestrian” OR “cyclist” OR “bicyclist” OR “bicycle” OR “motorcycle”). Although we found several studies that examined intersection-level or segment-level characteristics of the built environment and one that examined area level characteristics for one city in Latin America, we did not find any studies examining multiple cities or city-level characteristics of the built environment in relation to road-traffic mortality. In high-income settings, several studies have examined road-traffic mortality and specific characteristics of the built environment at the city level (eg, urban sprawl) and a few have examined urban design profiles of cities. These studies have, in general, shown that cities with urban developments or street designs that are more compact (eg, higher intersection density) tend to have lower road mortality, while those with higher rates tend to be more sprawling and have longer roads.

**Added value of this study**

To our knowledge, this is the first study to examine road-traffic mortality and city-level built environment characteristics across multiple cities and countries in Latin America. We found that across a diverse set of cities in low-income and middle-income countries cities with more compact development, higher population density, and with bus rapid transit had lower road-traffic mortality than cities with lower density development and population and without mass transit.

**Implications of all the available evidence**

Most research on predictors of road safety has focused on high-income countries. The findings of this study highlight the importance of city-level built environment characteristics for road safety in the rapidly growing cities of low-income and middle-income countries. Policies that promote higher density development, smaller block sizes, connectivity, and public transportation at the city level can help improve road safety, probably by improving the efficiency of traffic flow, reducing traffic speeds, and increasing walking, bicyling, and public transportation use.

The objective of this study was to examine patterns of road-traffic mortality in selected Latin American cities and to determine the association of mortality with city-level characteristics of the urban landscape, street design, transportation, population, and the social environment.

## Methods

### Sample

The main unit of analysis in this study was cities, as defined in the SALURBAL project (Salud Urbana en America Latina).^17^ Cities were selected for the study on the basis of their location in 11 countries and if they had a population of 100 000 or more. Each city was defined geographically by administrative units that encompassed the visually apparent urban extent (ie, the built-up area meaning the area of the city covered with buildings and other impervious surfaces), as of 2010, using satellite imagery overlaid with administrative boundaries. The SALURBAL study protocol was approved by the Drexel University Institutional Review Board with ID #1612005035 and by appropriate site-specific IRBs.

### Mortality

The primary outcome was road-traffic death rate among residents of SALURBAL cities. Mortality data were obtained from official country vital registry data and linked to SALURBAL-defined cities.^17^ Road-traffic deaths were identified using International Classification of Diseases version 10 (ICD-10) codes V01–V89 that describe road-traffic deaths (appendix p 15). We included all motor vehicle transportation-related deaths, whether considered to be traffic-related or non-traffic-related, because the small proportion non-traffic deaths (<1% of the total) could still capture traffic-related deaths in parking lots, driveways, private roads, parks, or other non-public road areas. Approximately 11% of deaths due to external causes in countries in the SALURBAL study were coded with ill-defined ICD-10 codes relevant to road-traffic deaths (appendix pp 4, 5). Some of these deaths are due to road-traffic collisions, but have not been specifically coded to road-traffic collision death codes; thus, we used well established methods from prior studies^18^, ^19^, ^20^ to redistribute ill-defined codes across specifically-defined and partially-defined codes using 100 multinomial draws, based on the observed distribution of road-traffic deaths by age (5-year age groups), sex, and country across all available years of deaths (appendix p 6). On average, across redistributions, 18·6% of road-traffic deaths originated from ill-defined causes, similar to what has been observed in country-level studies using similar methods (appendix pp 8, 9).^21^, ^22^, ^23^, ^24^ Counts of road-traffic deaths within each 5-year age–sex group were created for each redistribution by decedents’ city of residence and combining data for decedents aged 80 years or older (some countries combined population projections for those aged ≥80 years). We pooled data across years 2010–16 to create a more stable estimate for small age-by-sex cells. We used population post-censal projections or inter-censal estimations as denominators from national census bureaus or equivalent agencies to calculate road-traffic death rates per 100 000 population, by 5-year age–sex groups at the city level.^17^ We corrected population projections for the incomplete coverage of all deaths using an ensemble of death distribution methods.^25^, ^26^

Because cities were defined broadly, it is reasonable to assume that most traffic-related deaths in city residents occurred within the city boundaries. This approach also allows comparisons with national vital statistics data and is also consistent with other studies of factors associated with traffic deaths.^18^, ^19^

### Exposures

The primary exposures of interest were city-level built environment characteristics including spatial features of the urban landscape, street design, transportation, and population distribution based on prior literature, as well as hypothesised and theoretical understandings of these factors (appendix pp 16–17).^5^, ^6^, ^8^, ^9^, ^17^ Although few studies examined city-level, satellite-imagery-based urban landscape measures and road-traffic safety outcomes, we hypothesised that: higher fragmentation of urban development (patch density: density of separate built-up areas within city boundaries) and higher isolation of urban developments (area-weighted mean nearest neighbour distance between built-up patches) would have higher rates of road-traffic deaths. We also hypothesised that the higher the proportion of the city's geographic area that is built-up (ie, covered in buildings as determined by satellite imagery), the lower the road-traffic death rate.^17^ Street-design metrics included street connectivity (intersection density and average number of streets per node) and average street-segment length calculated from Open Street Maps.^17^ Previous research suggests that higher intersection density is associated with lower road-traffic mortality, whereas more complex intersections (ie, more connecting roads) and longer street lengths are associated with higher road-traffic mortality.^5^, ^6^, ^8^, ^9^ Transportation measures included the urban travel delay index, a measure of urban traffic congestion that reflects the amount of additional travel time during peak travel times compared to the average non-peak travel time, and the presence or absence of mass transit systems (bus rapid transit system or a rail transit system).^17^ Traffic congestion can reduce road-traffic mortality because of lower traffic speeds;^27^, ^28^ the effects of bus rapid transit or rail transit on road-traffic deaths have not been examined at the city-level across multiple cities, but the presence of such systems is expected to be beneficial to road safety.^29^, ^30^, ^31^ Population distribution measures included the population density within the built-up area of the city and population growth of the city during the years of the study (2010–16). A summary measure of the social environment (average of the *z*-score of the included variables) of the cities studied was constructed from each country's most recent census as of 2010. Variables used in the index include proportion of households with piped water in their dwelling, proportion of dwellings with a sewage network connection, proportion of households with overcrowding (defined as more than three people per room; values were reversed for index calculation), and proportion of adults aged 25 years or older who completed at least primary education. GDP for each city for 2010, in 2011 international US$, was derived from gridded GDP estimates.^32^, ^33^

### Statistical analysis

The 5-year age–sex road-traffic mortality rates were calculated for each city and each redistribution using the counts of the summed imputed road-traffic deaths divided by the corresponding population denominator derived from the summed 5-year age–sex group population projection. The average of the age-specific rates across redistributions were used to explore patterns and trends graphically in box plots and age trendlines by sex and country. We calculated direct age-standardised road-traffic mortality rates for each city using WHO 2000–25 standard population and 5-year age groups by sex and overall, and then averaged the age-standardised rate across the 100 redistributions.^34^ We used direct standardisation because there were no strata with fewer than 25 total deaths. Descriptive statistics of built and social city features were examined by quartiles of the averaged age-standardised rates. We compared our age-standardised rates to the WHO Global Status Report on Road Safety 2018 country-level mortality rates (crude and estimated)^2^ and Global Burden of Disease (GBD) 2019 mortality rates^35^ by pooling all deaths in the included cities by country to create a crude and age-standardised mortality rate for all cities in each country. We examined descriptive statistics of exposure variables by quartiles of city age-standardised rates and calculated Wald p values to examine differences between quartiles.

Associations between the outcome and exposures and covariates were evaluated using generalised linear mixed models (ie, multilevel) with a negative binomial distribution and robust variance and an offset for the population of each 5-year age–sex group. Models were adjusted for sex and age and included a random intercept for cities and a fixed effect for country. The ten cities in Central American countries (Costa Rica, El Salvador, Guatemala, and Panama) were treated as one category. Each redistribution was modelled separately, and coefficients and variances were extracted and then combined using Rubin's formula to produce rate ratios (RRs) and 95% CIs.^36^ City-level exposures were standardised to a mean of 0 and a SD of 1 except for presence or absence of mass transit. We first examined each exposure separately (single-exposure models) adjusted for age, sex, and country (model 1). Then, to determine whether any associations observed in model 1 were independent of each other, we fitted models that included all variables together (model 2). Lastly, we fitted a final model (model 3) in which we removed variables with very high Pearson's correlations (>0·9) or high variance inflation factors. We conducted sensitivity analyses repeating the main analyses and examining only cities with high quality road-traffic mortality coding (<20% of road-traffic deaths originating from ill-defined codes), including the proportion of road-traffic deaths from ill-defined codes as a covariate. We also conducted a complete case analysis using only deaths originally coded as road-traffic deaths and excluding ill-defined deaths from redistribution. We used StataMP (version 16.0) for descriptive and regression analyses, while the figures were created using ggplot2 in R (version 4.04).

### Role of the funding source

The funder had no role in study design, data collection, data analysis, data interpretation, or writing of the report.

## Results

**Figure 1:**
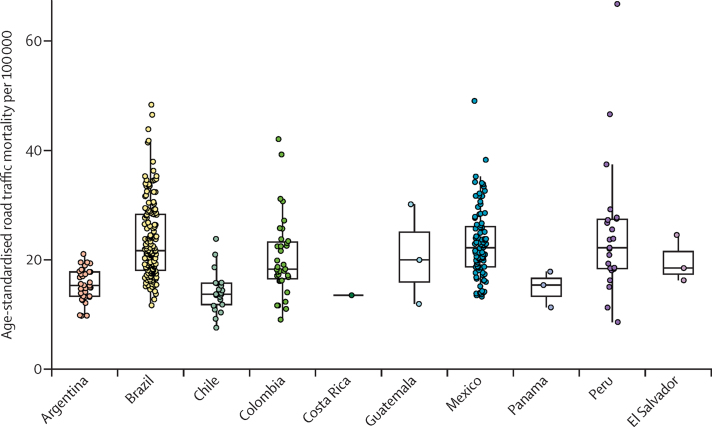
Age-standardised road traffic mortality per 100 000 population, by country

Road-traffic deaths from 366 cities in 10 countries were available for analysis. There were 328 408 road-traffic deaths over 1 918 741 080 person-years across all countries, with an average crude rate of 17·1 deaths per 100 000 person-years. Age-standardised rates varied across countries and substantially between cities within countries (table 1; figure 1). Across all countries, median road-traffic mortality rates ranged from 7·6 deaths per 100 000 population in Chile to 66·6 in Peru (appendix pp 17–25). The median of each country's age-standardised rates was comparable to country-level estimated total mortality rates in public reports by WHO^2^ and the GBD study,^35^ although Mexico and Peru had higher median rates than WHO and GBD country-level estimates (table 1).

**Table 1:** Age-standardised mortality rates of cities by country

	**Cities (N)**	**Years included in analysis**	**Mortality data administrative unit**	**Median age-standardised mortality per 100 000 population of SALURBAL cities**			**WHO estimate of road traffic mortality per 100 000 population at country level**^2^		**Global Burden of Disease estimate of road traffic mortality per 100 000 population at country level**^35^	
				Male	Female	Total	2010	2016	2010	2016
Argentina	33	2010–2016	Varies*	24·1 (21·6–28·8)	6·4 (5·9–6·9)	15·2 (13·3–17·7)	12·6	14·0	15·4 (14·9–15·9)	14·6 (14·0–15·1)
Brazil	152	2010–2016	Municipio	36·5 (30·1–47)	8·4 (6·7–10·6)	21·6 (18·0–28·3)	22·5	19·7	24·6 (24·0–25·2)	21·9 (21·3–22·5)
Chile	21	2010–2016	Comuna	21·6 (18·9–25·5)	5·9 (5·0–7·0)	13·7 (11·8–15·7)	12·3	12·5	14·4 (14·0–14·8)	13·1 (12·6–13·6)
Colombia	35	2010–2016	Municipio	30·6 (27·4–39·2)	7 (6·0–9·0)	18·1 (16·0–23·2)	15·6	18·5	16·7 (16·2–17·3)	16·1 (15·4–16·8)
Costa Rica	1	2010–2016	Cantones	20·9 (20·9–20·9)	6·1 (6·1–6·1)	13·5	12·7	16·7	16·4 (15·6–17·2)	18·9 (17·3–20·0)
El Salvador	3	2010–2014	Municipio	34·5 (28·8–42·5)	6·1 (5·7–10·4)	18·4 (16·2–24·5)	21·9	22·2	19·5 (18·1–24·6)	20·3 (16·2–26·6)
Guatemala	3	2010–2016	Municipio	34·1 (20·1–53·4)	8 (4·6–8·7)	19·9 (11·9–30·1)	6·7	16·6	14·0 (12·3–15·9)	16·1 (13·9–18·4)
Mexico	92	2010–2016	Municipio	35·0 (28·9–42·8)	10·0 (8–11·7)	22·1 (18·7–26·1)	14·7	13·1	17·8 (17·4–18·3)	16·6 (16·0–17·1)
Panama	3	2012–2016	Corregimiento	26·7 (18·3–28·7)	5·1 (4·6–7·2)	15·3 (11·3–17·8)	14·1	14·3	14·7 (13·8–15·4)	13·3 (12·6–14·2)
Peru	23	2010–2016	Distrito	32·4 (26·4–39·9)	11·3 (8·4–17·4)	22·2 (18·3–27·4)	15·9	13·5	17·4 (13·8–20·3)	14·3 (10·7–18·2)

Data are median (IQR) or mortality per 100 000 population (95% uncertainty interval).

* Comuna in the Ciudad Autonoma de Buenos Aires, partido in the province of Buenos Aires, departamento in the rest of the country.

**Figure 2:**
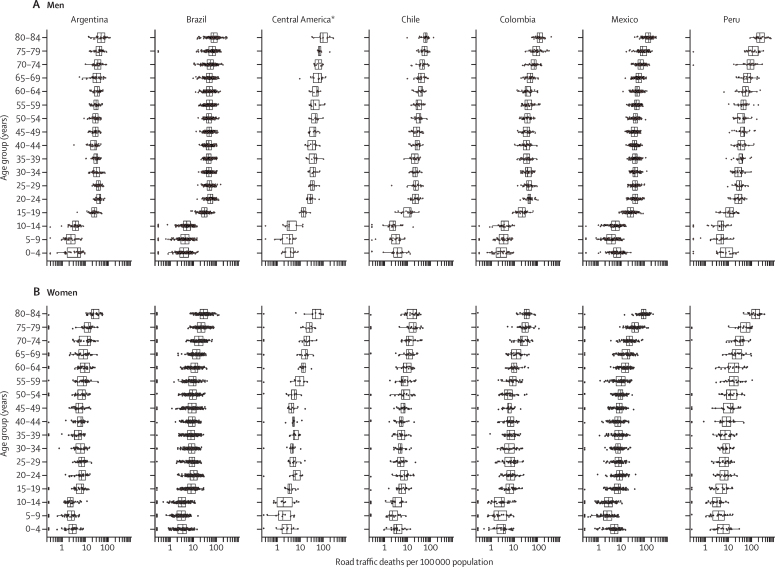
Road-traffic mortality per 100 000 population by country, sex, and age group (A) Road traffic mortality for men. (B) Road traffic mortality for women. *Includes Costa Rica, El Salvador, Guatemala, and Panama.

At country level, there were substantial differences in median age-standardised road-traffic mortality rates between men (21–36 deaths per 100 000 population) and women (5–11 deaths per 100 000 population). In Brazil and El Salvador, the greatest difference in rates between men and women was nearly 30 deaths per 100 000 population. These differences were also reflected in age-specific road-traffic mortality rates, which were higher for men than for women in all age groups (figure 2). Age-specific road-traffic mortality rates ranged from 0 to 698·8 deaths per 100 000 population across all cities (figure 2; appendix pp 10, 11). There were 369 age–sex groups with 0 deaths, mostly among women, those younger than 14 years old, and those aged 65–79 years. Nearly 50% of road-traffic deaths occurred among people younger than 35 years. In men, the age groups with the highest median road-traffic mortality rate per 100 000 population included men aged 65 years or older, whereas the age group with the lowest rate was boys aged 5–9 years (appendix p 12). Similar patterns were observed among women, with those aged 65 years and older having the highest median rate and girls aged 5–9 years old having the lowest median rate (appendix p 11).

Cities in the lowest quartile of age-standardised road-traffic mortality rates had the highest population density (median 6664 inhabitants/km^2^ [IQR 5537 to 9763]) and highest social environment index (0·42 [–0·04 to 0·70]) compared with cities in higher quartiles of age-standardised road-traffic mortality (table 2). The lowest quartile of mortality also had the highest proportion of the city that was built-up (5·2% [1·8 to 9·7]), the largest median distance between built-up areas (isolation, median 91·1 metres [IQR 85·6 to 98·9]), the highest intersection density (median 7·2 intersections per hectare [IQR 2·8 to 14·0]), the highest urban travel delay index (median 0·17 [0·11 to 0·30]), and the most cities with bus rapid transit or rail transportation systems (26 [29%] of 91). The highest quartile of road-traffic mortality had the highest annual population growth average (median 1323 per 100 000 population [IQR 835 to 7846] in 2010), lowest social environment index score (median –0·21 [IQR –0·55 to 0·11]), lowest per capita GDP in 2010 (median $10 903 in 2011 US$ [IQR 6653 to 16 676]), lowest patch density (median 0·41 per 100 hectares [IQR 0·19 to 0·68]), lowest proportion of built-up area (median 2·4% [IQR 1·2 to 4·7]), lowest intersection density (median 3·4 intersections per hectare [1·7 to 6·4]), and fewest cities with bus rapid transit or rail transportation systems (four [4%]).

**Table 2:** Quartiles of age-standardised road-traffic mortality per 100 000 population, by exposure

		**Quartile 17·6 to 16·7 (n=91)**	**Quartile 216·7 to 20·2 (n=92)**	**Quartile 320·2 to 25·5 (n=91)**	**Quartile 425·5 to 66·7 (n=92)**	Wald test p value*
Population distribution						
	Population density in built-up area (inhabitants per km^2^)†	6664 (5337 to 9763)	6281 (5148 to 8230)	5788 (4744 to 8234)	6056 (4691 to 7846)	0·5136
	Annual population growth average (average annual number of people added to the city per annual 100 000 population)†	1216 (885 to 1608)	1168 (892 to 1508)	1212 (876 to 1720)	1323 (835 to 1804)	0·7712
Social environment index		0·42 (−0·04 to 0·70)	0·30 (−0·19 to 0·52)	0·09 (−0·46 to 0·46)	−0·21 (−0·55 to 0·11)	0·0682
City gross domestic product (US$)†		14 729 (10 051 to 20 624)	14 729 (9848 to 20 046)	14 272 (8732 to 19 476)	10 903 (6653 to 16 676)	0·0186
Urban landscape						
	Patch density (patches per 100 hectares)	0·79 (0·37 to 1·52)	0·53 (0·28 to 1·07)	0·44 (0·25 to 0·85)	0·41 (0·19 to 0·68)	0·0482
	Area-weighted mean nearest neighbour (isolation, metres)	91·1 (85·6 to 98·9)	88·6 (82·6 to 95·2)	88·9 (82·4 to 94.6)	88 (82·9 to 94·8)	0·4342
	Proportion of city built up	5·2% (1·8 to 9·7)	4·1% (1·9 to 6·9)	2·9% (1·3 to 5·2)	2·4% (1·2 to 4·7)	0·1033
Street design						
	Intersection density (per hectare)	7·2 (2·8 to 14·0)	5·9 (2·8 to 9·4)	3·9 (1·8 to 7·4)	3·4 (1·7 to 6·4)	0·0970
	Average streets per node	2·9 (2·8 to 3·2)	3 (2·8 to 3·1)	3 (2·9 to 3·1)	3·1 (2·9 to 3·2)	0·3900
	Average street length (metres)	126 (112 to 154)	135 (121 to 166)	135 (118 to 159)	140 (123 to 180)	0·2866
Transportation						
	Urban travel delay index	0·17 (0·11 to 0·30)	0·12 (0·08 to 0·21)	0·11 (0·08 to 0·17)	0·1 (0·06 to 0·15)	0·1738
	Presence of bus rapid transit system or subway	26 (28·6%)	14 (15·4%)	9 (9·8%)	4 (4·4%)	0·0141

Data are median (IQR) or n (%).

* Tests if each quartile of age-standardised road mortality is equal to one another in a linear regression, with each covariate as the outcome and robust standard errors clustered by country.

† 2010 data.

Pearson correlations between the continuous city-level predictors were mostly small (<0·3) or moderate (0·3 to 0·6; appendix p 13). The only correlations greater than 0·6 (absolute value) were observed for proportion of the city built-up and intersection density (0·93), proportion of the city built-up and patch density (0·74), and patch density and intersection density (0·65).

**Figure 3:**
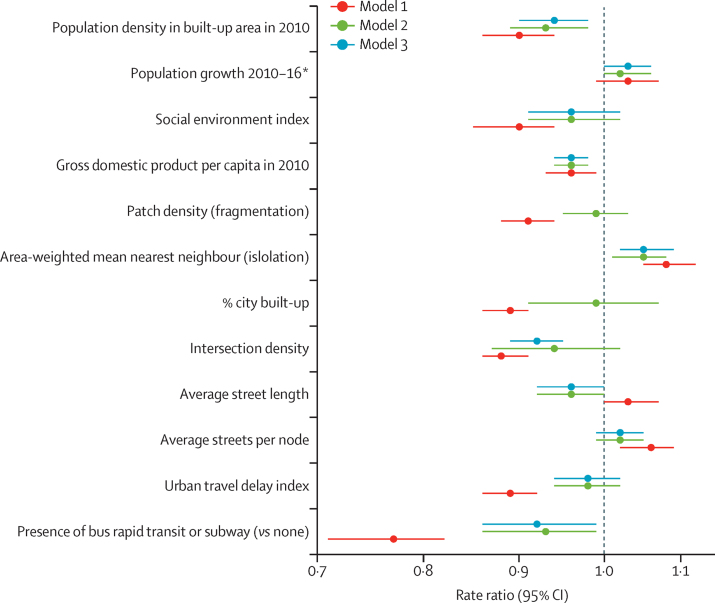
Forest plot of the association of road-traffic mortality in single exposure and multivariable models Rate ratios and 95% CI CIs were estimated in mixed-effects generalised linear model regression with negative binomial distribution and robust standard errors. All RRs and 95% CIs reflect a difference of 1 SD except bus rapid transit or subway. All models are adjusted for a fixed effect for country, sex, and 5-year age group. Model 1 is a single exposure model of each exposure and covariate. Model 2 includes all exposures and covariates in a multivariable model. Model 3 removed patch density and proportion of city built-up from the model due to high correlation with other variables. *Measured for 2010–14 in El Salvador and 2012–16 in Panama to match the years of outcome data available.

In single-exposure models adjusted for country (fixed effect), sex, and age group (model 1), most measures exhibited strong associations with road-traffic deaths (figure 3; table 3). Higher population density, higher social environment index, higher GDP per capita, higher development fragmentation, higher proportion of the city area that was built-up, higher intersection density, and a higher urban travel delay index were associated with lower road-traffic mortality; a 1 SD higher value for these metrics is associated with a 11–12% lower rate (table 3). Road-traffic mortality was lower in cities with a bus rapid transit or subway system than those without (RR 0·77 [95% CI 0·71–0·82]). By contrast, cities with faster population growth, higher isolation of built-up areas, and more average streets per node had higher road-traffic mortality rates than did cities with lower values; these metrics were associated with a 3–8% higher rate per 1 SD (table 3).

**Table 3:** Association between road traffic mortality and exposures, by model

		**Model 1**	**Model 2**	**Model 3**
Population measures				
	Population density in built-up area, 2010	0·90 (0·86–0·94)	0·93 (0·89–0·98)	0·94 (0·90–0·98)
	Population growth 2010–16*	1·03 (0·99–1·07)	1·02 (1·00–1·06)	1·03 (1·00–1·06)
Social environment index		0·90 (0·85–0·94)	0·96 (0·91–1·02)	0·96 (0·91–1·02)
Gross domestic product per capita in 2010		0·96 (0·93–0·99)	0·96 (0·94–0·98)	0·96 (0·94–0·98)
Urban landscape				
	Patch density (fragmentation)	0·91 (0·88–0·94)	0·99 (0·95–1·03)	..
	Area-weighted mean nearest neighbour (isolation)	1·08 (1·05–1·12)	1·05 (1·01–1·08)	1·05 (1·02–1·09)
	Proportion of city built up	0·89 (0·86–0·91)	0·99 (0·91–1·07)	..
Road network measures				
	Intersection density	0·88 (0·86–0·91)	0·94 (0·87–1·02)	0·92 (0·89–0·95)
	Average street length (metres)	1·03 (1·00–1·07)	0·96 (0·92–1·00)	0·96 (0·92–1·00)
	Average streets per node	1·06 (1·02–1·09)	1·02 (0·99–1·05)	1·02 (0·99–1·05)
Transportation measures				
	Urban travel delay index	0·89 (0·86–0·92)	0·98 (0·94–1·02)	0·98 (0·94–1·02)
	Presence of bus rapid transit system or subway (*vs* none)	0·77 (0·71–0·82)	0·93 (0·86–0·99)	0·92 (0·86–0·99)

Data are rate ratio (95% CI). All models are adjusted for a fixed effect for country, sex, and 5-year age group. Model 1 is a single exposure model of each exposure and covariate. Model 2 includes all exposures and covariates in a multivariable model. Model 3 includes all exposures and covariates except patch density and percent built-up area. Rate ratios were estimated using mixed-effects generalised linear model regression with negative binomial distribution and robust standard errors. All rate ratios and 95% CIs reflect a difference of 1 SD except for bus rapid transit system or subway.

* Measured for 2010–14 in El Salvador and 2012–16 in Panama to match the years of outcome data available.

In multivariable models (model 2), higher population density, higher GDP per capita, and presence of bus rapid transit or subway systems remained significantly associated with lower traffic mortality, and higher isolation remained associated with higher mortality (table 3). Higher social environment index and higher intersection density were also associated with lower rates, but these associations were no longer statistically significant after adjustment. Population growth, patch density, proportion of city built-up, average streets per node, and urban travel delay index were not significantly associated with mortality rates after adjustment. The association between average street length and mortality became inverse compared with model 1, but was not statistically significant.

Given the very high correlation between proportion of city built-up and intersection density (0·93), we fit a final model, removing proportion of city built-up (model 3; table 3). Patch density was also removed from this final model because its coefficient cannot be properly interpreted if proportion of city built-up is not in the model. Results were similar to those in model 2: higher population density, higher GDP per capita, and the presence of bus rapid transit or subway systems all remained significantly associated with lower road-traffic mortality, and higher isolation was associated with higher road-traffic mortality. In this model, higher intersection density was also associated within lower road-traffic mortality.

Sensitivity analyses that excluded cities with poor death coding (>20% road-traffic deaths originating from ill-defined codes; n=152) had similar results to the main analyses, albeit with wider 95% CIs for some covariates (appendix, p 14). Including the proportion of ill-defined deaths as a covariate in analyses did not change the results substantially from the main analysis. A complete case analysis excluding redistributed ill-defined codes also resulted in similar findings to the main analyses, but with wider 95% CIs for some exposures (appendix p 14).

## Discussion

In this study, we used a heterogeneous sample of 366 Latin American cities and mortality data derived from official vital registry data between 2010 and 2016 to examine city-level built environment factors and road-traffic mortality. In multivariable adjusted models, Latin American cities with higher population density, higher intersection density, and those with a mass transit system such as a subway or bus rapid transit system had significantly lower road-traffic death rates than cities with lower values of these characteristics (or absence). Cities with higher GDP per capita also had lower road-traffic mortality. By contrast, cities with more isolated built-up areas had significantly higher road-traffic mortality. This study also showed that there is substantial heterogeneity between Latin American cities in terms of road-traffic mortality, both within and between countries.

Population density and several urban form measures were associated with road-traffic mortality. Higher population density in urban areas is typically linked with higher levels of walking and public transportation use and less driving,^37^ and was consistently associated with lower traffic mortality in our study, even after adjustment for other factors examined. Studies examining population density in relationship to road-traffic deaths have found conflicting results, likely due to differences in the geographic level being examined and other covariates included in the models.^38^, ^39^, ^40^ Many of the studies showing that higher population density is associated with higher traffic related mortality are at the province or country-level, probably reflecting urban–rural differences.^38^, ^40^ By contrast, most studies at the city or sub-city-level have shown that areas with a higher population density typically have a lower road-traffic mortality rate per population or vehicle miles travelled.^27^, ^40^, ^41^

Higher fragmentation (ie, more disconnected built-up areas) and higher proportion of the city area that was built up were also associated with lower traffic mortality, while higher urban development isolation was associated with higher traffic mortality. The association of greater isolation with higher traffic mortality persisted after adjustment for other factors. Isolation is the average distance among disconnected patches of urban development in the city's geography, thus a higher value indicates that patches of development are, on average, further apart from each other. Cities with this type of development could be expected to have higher road-traffic mortality because residents must traverse longer distances to get places, facing lower road connectivity or high-volume roads near residential areas, and possibly high-speed roads in between developments.^11^

City street connectivity (eg, intersection density) has been associated with road-traffic mortality in previous studies, with more connected cities having lower road-traffic mortality,^6^, ^11^, ^42^, ^43^ but few of these studies focused on low-income and middle-income regions. We showed the potential effect of street features on traffic-related mortality across a large set of Latin American cities, a context of high urbanisation and high traffic-related mortality. Consistent with previous work,^28^, ^42^ we found that higher intersection density at the city level was associated with lower death rates across all models. Greater intersection density is hypothesised to affect risk of road-traffic death primarily via motor vehicle speeds, by slowing down motorists on denser networks.^10^, ^28^, ^42^, ^44^ When analysed separately from other factors, the presence of longer streets was associated with higher traffic-related mortality (though not significantly), which is consistent with previous reports, suggesting this association could be due to motorists being able to reach higher speeds on longer roads.^44^ After adjustment for other factors, cities with longer streets had slightly lower road-traffic death rates than did cities with shorter streets in our analyses, but confidence intervals just barely included the null. Although this finding contradicts several previous studies^28^, ^45^, ^46^, ^47^ at least one study at the city level is partly consistent with our results.^6^ That study showed that cities with longer high-speed roads (highways) per inhabitant had higher passenger vehicle fatality rates, whereas cities with longer surface roads (arterials) per inhabitant had lower passenger vehicle fatality rates.^6^ The relation of road length with road-traffic deaths deserves further exploration in low-income and middle-income cities.

Our findings indicate that cities with bus rapid transit or passenger railway systems had a lower rate of road-traffic deaths than did cities without those systems. The association was reduced but persisted after adjustment for the other factors. Previous studies of specific cities had mixed results, all focusing on within-city changes in a single city in road-traffic collisions.^30^ To date, none have examined as many cities as in this study or compared cities with and without bus rapid transit.^30^ Mass transit modes have lower rates of road-traffic collisions and deaths than any other mode of transportation, and higher city bus use might also result in fewer cyclist and pedestrian deaths.^29^, ^31^, ^48^ As more people use these modes, there are fewer motorists and thus fewer opportunities for collisions to happen. These systems also tend to produce higher volumes of pedestrians around stations and stops, which could result in a safety-in-numbers effect by slowing motor vehicle traffic.^7^ At least 170 cities worldwide have implemented bus rapid transit, which originated in Curitiba Brazil in 1974.^30^ Our results provide additional support to the implementation of these types of mass transit systems for road safety in addition to their benefits as sustainable transportation.

A better city-level social environment (in terms of social environment index) was associated with lower traffic mortality, similar to what has been observed in a previous study,^49^ though when adjusting for city-level GDP, city-level social environment was no longer statistically significant. Previous studies examining the association of GDP with road-traffic deaths showed lower rates in cities with higher GDP, though increases in GDP have been associated with more traffic deaths as a result of the acceleration of economic activity.^50^, ^51^, ^52^ The associations of higher socioeconomic characteristics or GDP with lower rates (cross-sectionally) could reflect several processes occurring in cities with better socioeconomic conditions, such as improved quality of streets and vehicles, increased access to emergency services and medical care, and the development of public transportation.^51^

Strengths of our study include the use of harmonised data drawn from the city level across a broad range of diverse cities across Latin America. The ecological design is appropriate given inferences at the city level rather than individual level. Ideally, city-level studies would use deaths occurring within the boundaries of the city (possibly excluding intercity highways) and a measure of traffic volume (and possibly also pedestrian traffic) as denominators. Unfortunately, such data were not available across all cities in our study. However, we believe that given the broad definition of cities that we used (and the fact that available data suggests that 80% of deaths to residents were coded as occurring within the city boundaries), death rates in residents are a reasonable outcome to focus on. However, measurement error attributable to this approximation could have hampered our ability to detect some city-level associations with road-traffic deaths.

Misclassification of road-traffic deaths is a potential limitation; however, we used well accepted methods to redistribute ill-defined causes of death to address potential bias from using just one redistribution. Sensitivity analyses showed that the associations between exposures and road-traffic mortality did not change substantially. The correction factors for incomplete registration are imperfect at the city level and could either underestimate or overestimate mortality in some cities. Improving the registration and the coding of mortality data in the region remains an important priority. We did not account for potential differences between cities in terms of transportation use frequency, such as number of trips or distance travelled due to the unavailability of these measures in many Latin American cities. Some of the exposures we examined might have changed during the 2010–16 period we examined; thus, future longitudinal studies might be able to examine temporal trends as data improve. Finally, we examined several intercorrelated variables and, while, correlations were not very high, we removed variables that were most highly correlated. Many of these variables capture closely related domains (eg, fragmentation and isolation) and can also act synergistically, which might result in adjusted results underestimating the potential effect of these factors on traffic deaths.

Road-traffic mortality in Latin American cities is associated with specific city-level attributes, suggesting that broad urban planning policies can be relevant to achieving Sustainable Development Goal 3.6 of halving the number of global deaths and injuries from road-traffic accidents. Well designed and well implemented policies addressing street-level and behavioural factors can have substantial effects on road-traffic mortality (eg, mitigating dangerous intersection or drunk-driving enforcement), but the effects might not be sustainable. By contrast, urban form, street design, and public transportation policies (factors examined in this study), can potentially reduce road-traffic deaths in the long term across an entire city.^10^, ^53^ Hence, although effect sizes appear small, the effect of the factors we studied could be large over time. Our results indicate that the urban landscape, street network design, and public transportation ought to be considered in future urban design and transport policies with an eye towards more comprehensively improving road safety, such as Vision Zero, Complete Streets, and other road safety initiatives. The findings of this study provide evidence for city planners, traffic engineers, policymakers, and community members to consider how their cities are developing and what the effect of city-level built environment factors might be on road-traffic safety. Aiming to design more connected cities and increasing public transportation options could help improve road-traffic safety in low-income and middle-income countries, while also potentially providing other health benefits by increasing physical activity and reducing air pollution caused by personal motor vehicle use.

## Data sharing

The SALURBAL project welcomes queries from anyone interested in learning more about its dataset and potential access to data. To learn more about SALURBAL's dataset, visit the SALURBAL project website or contact the project at salurbal@drexel.edu. After publication of this study, study protocols, data dictionaries, and requested study data may be made available to interested investigators after they have signed a data use agreement with SALURBAL and if their study proposal, developed in collaboration with SALURBAL investigators, is approved by the SALURBAL proposal and publications committee. Some data may not be available to external investigators because of data confidentiality agreements.

## Declaration of interests

We declare no competing interests.

## References

[1] Institute for Health Metrics and Evaluation (2014).

[2] WHO (2018).

[3] Villaveces A, Sanhueza A, Henríquez Roldán CF, Escamilla-Cejudo JA, Rodrigues EMS (2021). Transport modes and road traffic mortality in the Americas: deaths among pedestrian and motorcycle users through the lifespan. Int J Inj Contr Saf Promot.

[4] Pan American Health Organization (2013).

[5] Ewing R, Dumbaugh E (2009). The built environment and traffic safety: a review of empirical evidence. J Plan Lit.

[6] Moeinaddini M, Asadi-Shekari Z, Zaly Shah M (2014). The relationship between urban street networks and the number of transport fatalities at the city level. Saf Sci.

[7] Jacobsen PL, Ragland DR, Komanoff C (2015). Safety in numbers for walkers and bicyclists: exploring the mechanisms. Inj Prev.

[8] Stoker P, Garfinkel-Castro A, Khayesi M (2015). Pedestrian safety and the built environment: a review of the risk factors. J Plan Lit.

[9] Ameratunga S, Hijar M, Norton R (2006). Road-traffic injuries: confronting disparities to address a global-health problem. Lancet.

[10] Stevenson M, Thompson J, de Sá TH (2016). Land use, transport, and population health: estimating the health benefits of compact cities. Lancet.

[11] Thompson J, Stevenson M, Wijnands JS (2020). A global analysis of urban design types and road transport injury: an image processing study. Lancet Planet Health.

[12] Donroe J, Tincopa M, Gilman RH, Brugge D, Moore DAJ (2008). Pedestrian road traffic injuries in urban Peruvian children and adolescents: case control analyses of personal and environmental risk factors. PLoS ONE.

[13] de Andrade L, Vissoci JRN, Rodrigues CG (2014). Brazilian road traffic fatalities: a spatial and environmental analysis. PLoS One.

[14] Fox L, Serre ML, Lippmann SJ (2014). Spatiotemporal approaches to analyzing pedestrian fatalities: the case of Cali, Colombia. Traffic Inj Prev.

[15] Quistberg DA, Koepsell TD, Boyle LN, Miranda JJ, Johnston BD, Ebel BE (2014). Pedestrian signalization and the risk of pedestrian-motor vehicle collisions in Lima, Peru. Accid Anal Prev.

[16] Global SDG Indicator Platform (Oct 31, 2018). 3.6.1 Death rate due to road traffic injuries. https://sdg.tracking-progress.org/indicator/3-6-1-death-rate-due-to-road-traffic-injuries.

[17] Quistberg DA, Diez Roux AV, Bilal U (2019). Building a data platform for cross-country urban health studies: the SALURBAL study. J Urban Health.

[18] Bhalla K, Naghavi M, Shahraz S, Bartels D, Murray CJL (2009). Building national estimates of the burden of road traffic injuries in developing countries from all available data sources: Iran. Inj Prev.

[19] Bhalla K, Shahraz S, Bartels D, Abraham J (2009). Methods for developing country level estimates of the incidence of deaths and non-fatal injuries from road traffic crashes. Int J Inj Contr Saf Promot.

[20] Pérez-Núñez R, Mojarro-Íñiguez MG, Mendoza-García ME, Rosas-Osuna SR, Híjar M (2016). Underestimation of mortality caused by traffic in Mexico: an analysis at the subnational level. Salud Pública Méx.

[21] Johnson SC, Cunningham M, Dippenaar IN (2021). Public health utility of cause of death data: applying empirical algorithms to improve data quality. BMC Med Inform Decis Mak.

[22] Bhalla K, Gleason K (2020). Effects of vehicle safety design on road traffic deaths, injuries, and public health burden in the Latin American region: a modelling study. Lancet Glob Health.

[23] Bhalla K, Harrison JE (2015). GBD-2010 overestimates deaths from road injuries in OECD countries: new methods perform poorly. Int J Epidemiol.

[24] Bhalla K, Harrison JE, Shahraz S, Fingerhut LA (2010). Availability and quality of cause-of-death data for estimating the global burden of injuries. Bull World Health Organ.

[25] Ho HC, Wong MS, Yang L (2018). Spatiotemporal influence of temperature, air quality, and urban environment on cause-specific mortality during hazy days. Environ Int.

[26] Bilal U, Alazraqui M, Caiaffa WT (2019). Inequalities in life expectancy in six large Latin American cities from the SALURBAL study: an ecological analysis. Lancet Planety Health.

[27] Elvik R (2009). The non-linearity of risk and the promotion of environmentally sustainable transport. Accid Anal Prev.

[28] Mohan D, Bangdiwala SI, Villaveces A (2017). Urban street structure and traffic safety. J Safety Res.

[29] Stimpson JP, Wilson FA, Araz OM, Pagan JA (2014). Share of mass transit miles traveled and reduced motor vehicle fatalities in major cities of the United States. J Urban Health.

[30] Vecino-Ortiz AI, Hyder AA (2015). Road safety effects of bus rapid transit (BRT) systems: a call for evidence. J Urban Health.

[31] Morency P, Strauss J, Pépin F, Tessier F, Grondines J (2018). Traveling by bus instead of car on urban major roads: safety benefits for vehicle occupants, pedestrians, and cyclists. J Urban Health.

[32] Kummu M, Taka M, Guillaume JHA (2018). Gridded global datasets for gross domestic product and Human Development Index over 1990–2015. Sci Data.

[33] Gennaioli N, La Porta R, Lopez-de-Silanes F, Shleifer A (2012). Human capital and regional development. Q J Econ.

[34] Ahmad OB, Boschi-Pinto C, Lopez AD, Murray CJ, Lozano R, Inoue M (2001).

[35] Global Burden of Disease Collaborative Network (2020). http://ghdx.healthdata.org/gbd-results-tool.

[36] Little RJA, Rubin DB (2020).

[37] Barrington-Leigh C, Millard-Ball A (2020). Global trends toward urban street-network sprawl. Proc Natl Acad Sci USA.

[38] Spoerri A, Egger M, von Elm E (2011). Mortality from road traffic accidents in Switzerland: longitudinal and spatial analyses. Accident Anal Prev.

[39] Gedeborg R, Thiblin I, Byberg L, Melhus H, Lindbäck J, Michaelsson K (2010). Population density and mortality among individuals in motor vehicle crashes. Inj Prev.

[40] Dumbaugh E, Rae R (2009). Safe urban form: revisiting the relationship between community design and traffic safety. J Am Plann Association.

[41] Yañez-Pagans P, Martinez D, Mitnik OA, Scholl L, Vazquez A (2019). Urban transport systems in Latin America and the Caribbean: lessons and challenges. Lat Am Econ Rev.

[42] Ewing R, Hamidi S, Grace JB (2014). Urban sprawl as a risk factor in motor vehicle crashes. Urban Stud.

[43] Ewing R, Schieber RA, Zegeer CV (2003). Urban sprawl as a risk factor in motor vehicle occupant and pedestrian fatalities. Am J Public Health.

[44] Loder A, Ambühl L, Menendez M, Axhausen KW (2019). Understanding traffic capacity of urban networks. Sci Rep.

[45] Wier M, Weintraub J, Humphreys EH, Seto E, Bhatia R (2009). An area-level model of vehicle-pedestrian injury collisions with implications for land use and transportation planning. Accident Anal Prevn.

[46] Ewing R, Hamidi S, Grace JB (2016). Urban sprawl as a risk factor in motor vehicle crashes. Urban Stud.

[47] Ukkusuri S, Miranda-Moreno LF, Ramadurai G, Isa-Tavarez J (2012). The role of built environment on pedestrian crash frequency. Saf Sci.

[48] Jacobsen PL, Ragland DR, Komanoff C (2015). Safety in numbers for walkers and bicyclists: exploring the mechanisms. Inj Prev.

[49] Morency P, Gauvin L, Plante C, Fournier M, Morency C (2012). Neighborhood social inequalities in road traffic injuries: the influence of traffic volume and road design. Am J Public Health.

[50] Bishai D, Quresh A, James P, Ghaffar A (2006). National road casualties and economic development. Health Econ.

[51] Paulozzi LJ, Ryan GW, Espitia-Hardeman VE, Xi YL (2007). Economic development's effect on road transport-related mortality among different types of road users: a cross-sectional international study. Accident Ana Prev.

[52] Yannis G, Papadimitriou E, Folla K (2014). Effect of GDP changes on road traffic fatalities. Saf Sci.

[53] Salmon PM, McClure R, Stanton NA (2012). Road transport in drift? Applying contemporary systems thinking to road safety. Saf Sci.

