# Supplementary material for: Urban landscape and street-design factors associated with road-traffic mortality in Latin America between 2010 and 2016 (SALURBAL): an ecological study
Source: Lancet Planet Health. 2022 Feb 9;6(2):e122–31. doi: 10.1016/S2542-5196(21)00323-5 (PMC8850369; doi:10.1016/S2542-5196(21)00323-5)
Supplement: Supplementary appendix [file mmc1.pdf]

THE LANCET
Planetary Health

Supplementary appendix

This appendix formed part of the original submission and has been peer reviewed. We post it as supplied by the authors.

Supplement to: Quistberg DA, Hessel P, A Rodriguez DA, et al. Urban landscape and street-design factors associated with road-traffic mortality in Latin America between 2010 and 2016 (SALURBAL): an ecological study. *Lancet Planet Health* 2022; **6:** e122–31.

Urban Landscape and Street Design Factors Associated with Road Traffic Mortality: A study of 328,408 road traffic deaths in 366 Latin American Cities

Supplementary Material

Please contact the corresponding author with any questions:

Dr. Alex Quistberg
Urban Health Collaborative
Drexel University
daq26@drexel.edu

Table of Contents

Appendix I: Supplementary Tables and Figures

1) **Supplementary Figure 1:** Distribution of defined and ill-defined ICD10 codes by country
2) **Supplementary Table 1:** Redistribution schema of ill-defined mortality ICD10 codes
3) **Supplementary Table 2:** Descriptive statistics of redistributed ill-defined codes
4) **Supplementary Figure 2:** Proportion of road-traffic deaths in cities originating from ill-defined codes by country
5) **Supplementary Figure 3:** Proportion of road-traffic deaths originating from ill-defined codes by city and country
6) **Supplementary Table 3:** Descriptive statistics of age-specific groups by sex and country
7) **Supplementary Table 4:** Pearson correlation matrix of exposure variables.
8) **Supplementary Table 5:** Sensitivity Analyses Results

Appendix II: Description of specific ICD-10 codes used to identity road-traffic deaths
Appendix III: Descriptions of exposure measures and their interpretation
Appendix IV: Road-Traffic Mortality Rates, 2010-2016 per 100,000 Population for 366 Cities in Latin America

Supplementary Figure 1. Distribution of external cause ICD10 codes by country before any redistribution.

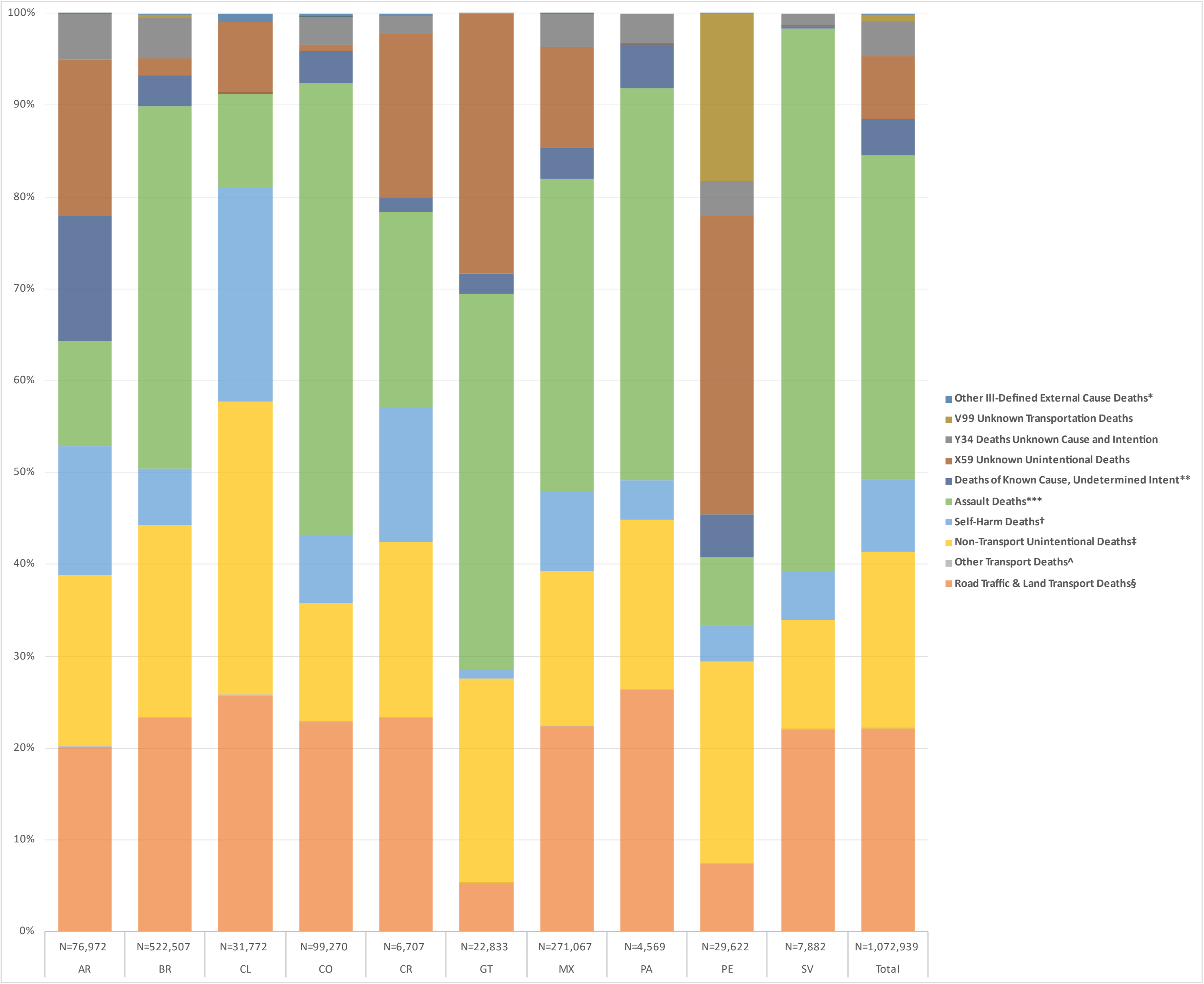

* ICD10 Codes Y872 (Sequelae of events of undetermined intent), Y899 (Sequelae of unspecified external cause), Y859 (Sequelae of other and unspecified transport injuries), Y32 (Crashing of motor vehicle, undetermined intent)

** ICD10 Codes Y10-Y31, Y33 (Event of Undetermined Intent)

*** ICD10 Codes X85-Y02, Y04-Y09, Y35-Y36, Y871, Y890, Y891

† ICD10 Codes X60-X81, X83-X84, Y870

‡ ICD10 Codes W00-X58, Y40-Y84, Y86, Y88

^ ICD10 Codes V90-V98

§ ICD10 Codes (V01-V89, Y850)

Supplementary Table 1: Redistribution schema of ill-defined mortality codes relevant to road traffic deaths by specifically coded or partially coded external causes of death.

			Specific and Partial External Causes of Death						
			V01-V89, Y850	V90-V98	W00-X58, Y40-Y84, Y86, Y88	X60-X81, X83-X84, Y870	X85-Y02, Y04-Y09, Y35-Y36, Y871, Y890, Y891	X82	Y03
			Land Transport Injuries	Other Transport Injuries	Other Unintentional Injuries	Self Harm Injuries	Intentional Injuries	Self Harm by motor vehicle	Assault by motor vehicle
Ill-defined Causes to be Redistributed	Y34	Unspecified event, undetermined intent	Yes	Yes	Yes	Yes	Yes	Yes	Yes
	Y872	Sequelae of events of undetermined intent	Yes	Yes	Yes	Yes	Yes	Yes	Yes
	Y899	Sequelae of unspecified external cause	Yes	Yes	Yes	Yes	Yes	Yes	Yes
	X59	Ill-defined unintentional injuries	Yes	Yes	Yes	N/A	N/A	N/A	N/A
	Y859	Sequelae of other and unspecified transport injuries	Yes	Yes	N/A	N/A	N/A	N/A	N/A
	V99	Ill-defined transportation injuries	Yes	Yes	N/A	N/A	N/A	N/A	N/A
	Y32	Crashing of motor vehicle, undetermined intent	Yes	N/A	N/A	N/A	N/A	Yes	Yes

Supplementary Table 2: Redistribution results of ill-defined mortality codes. Mean, standard deviation, minimum and maximum proportion of codes redistributed to each specific-defined group of code.

	Sequelae of events of undetermined intent (Y872)	Sequelae of unspecified external cause (Y899)	Unspecified event, undetermined intent (Y34)	Ill-defined unintentional injuries (X59)	Sequelae of other and unspecified transport injuries (Y859)	Ill-defined transportation injuries (V99)	Crashing of motor vehicle, undetermined intent (Y32)
	**N=704**	**N=684**	**N=41,148**	**N=73,456**	**N=166**	**N=7,193**	**N=77**
	**Mean (SD) [Min, Max]**	**Mean (SD) [Min, Max]**	**Mean (SD) [Min, Max]**	**Mean (SD) [Min, Max]**	**Mean (SD) [Min, Max]**	**Mean (SD) [Min, Max]**	**Mean (SD) [Min, Max]**
**Land Transport Injuries (V01-V89, Y850)**	28.8 (13.9) [0, 58]	31.2 (7.8) [5, 65]	34.6 (9.7) [5, 73]	48.9 (17.8) [4, 90]	99.3 (0.9) [97, 100]	98.8 (1.2) [91, 100]	99.9 (0.31) [99, 100]
**Other Transport Injuries (V90-V98)**	0.14 (0.39) [0, 2]	0.24 (0.51) [0, 3]	0.22 (0.5) [0, 7]	0.31 (0.63) [0, 7]	0.5 (0.73) [0, 3]	0.97 (1.1) [0, 8]	0.1 (0.31) [0, 1]
**Other Unintentional Injuries (W00-X58, Y40-Y84, Y86, Y88)**	19.2 (15.1) [0, 68]	38.3 (18.8) [3, 87]	28.1 (17.1) [0, 89]	50.8 (17.9) [10, 96]	0.18 (0.45) [0, 2]		
**Self-Harm (X60-X81, X83-X84, Y870)**	6.7 (4.2) [0, 24]	17.1 (10.8) [0, 46]	10.0 (6.9) [0, 42]				
**Assaults (X85-Y02, Y04-Y09, Y35-Y36, Y871, Y890, Y891)**	31.0 (21.9) [0, 72]	9.7 (13.0) [0, 64]	22.7 (15.9) [0, 81]				
**MV Self Harm (X82)**	0 (0) [0, 0]	0 (0) [0, 0]	0 (0) [0, 0]				0 (0) [0, 0]
**MV Homicides (Y03)**	6.4 (4.3) [0, 17]	3.4 (3.1) [0, 21]	4.4 (3.1) [0, 22]				0 (0) [0, 0]

Supplementary Figure 2. Proportion of road traffic deaths in cities by country originating from ill-defined codes, 2010-2016.

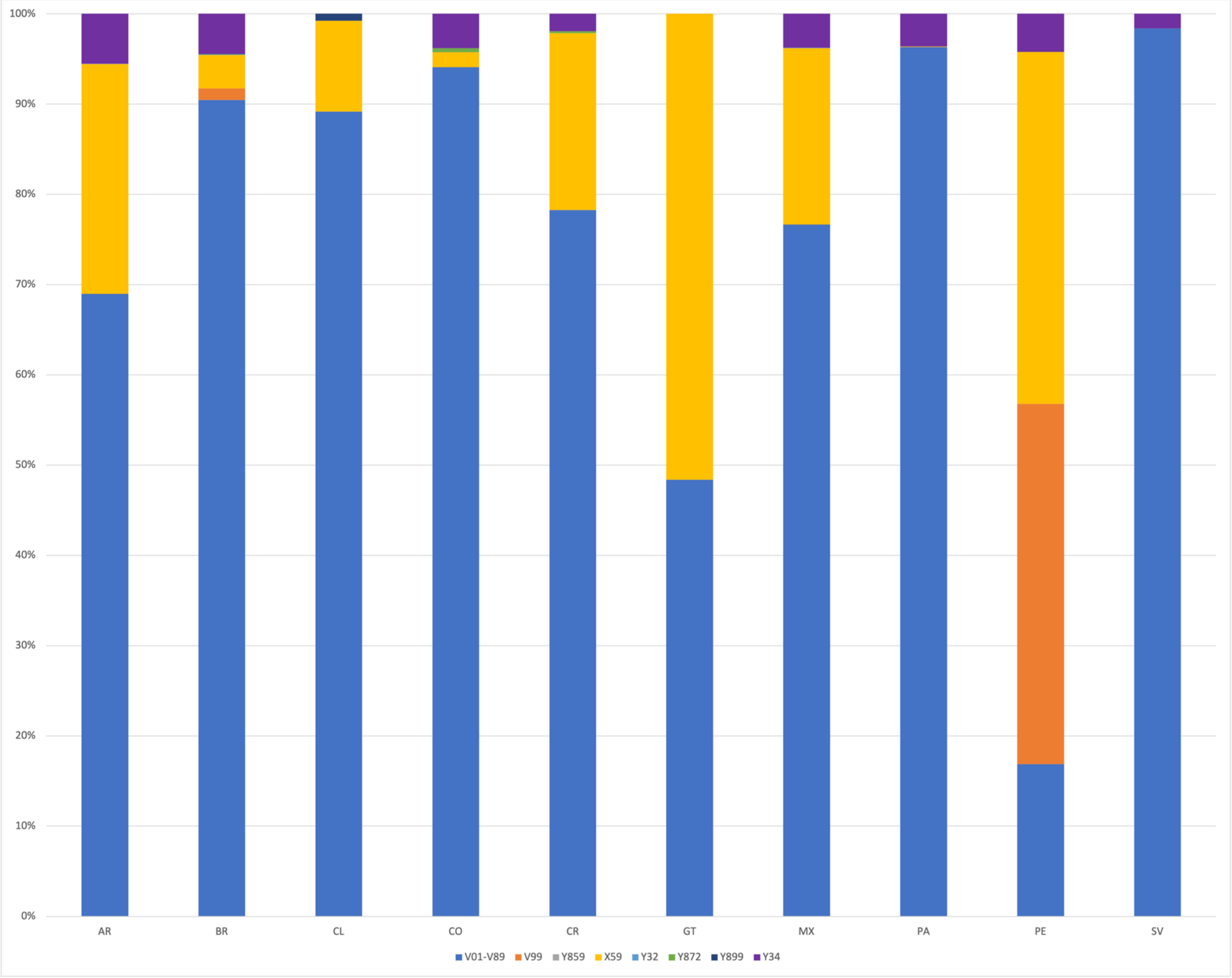

Supplementary Figure 3. Proportion of road traffic deaths originating from each ill-defined code by city.
A) Argentina, Chile, Colombia and Peru

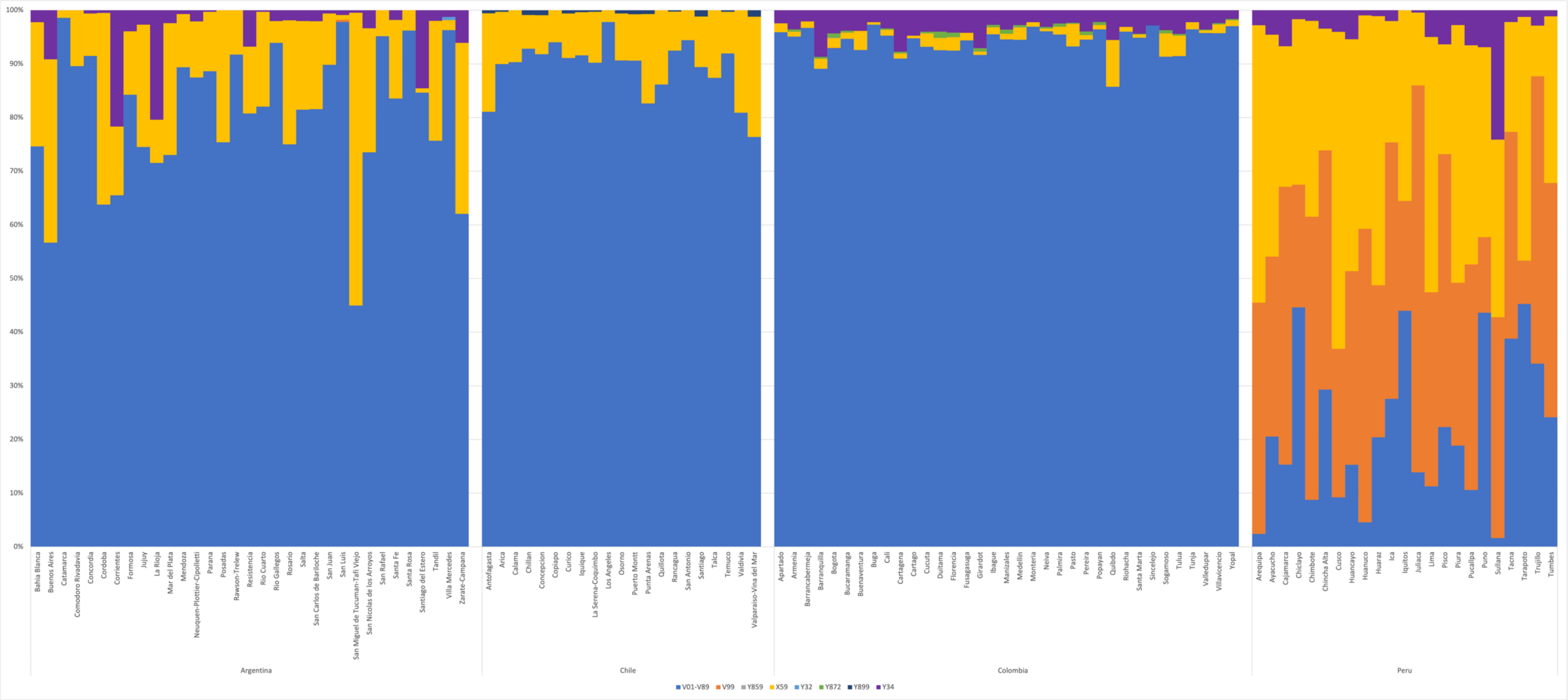

B) Brazil

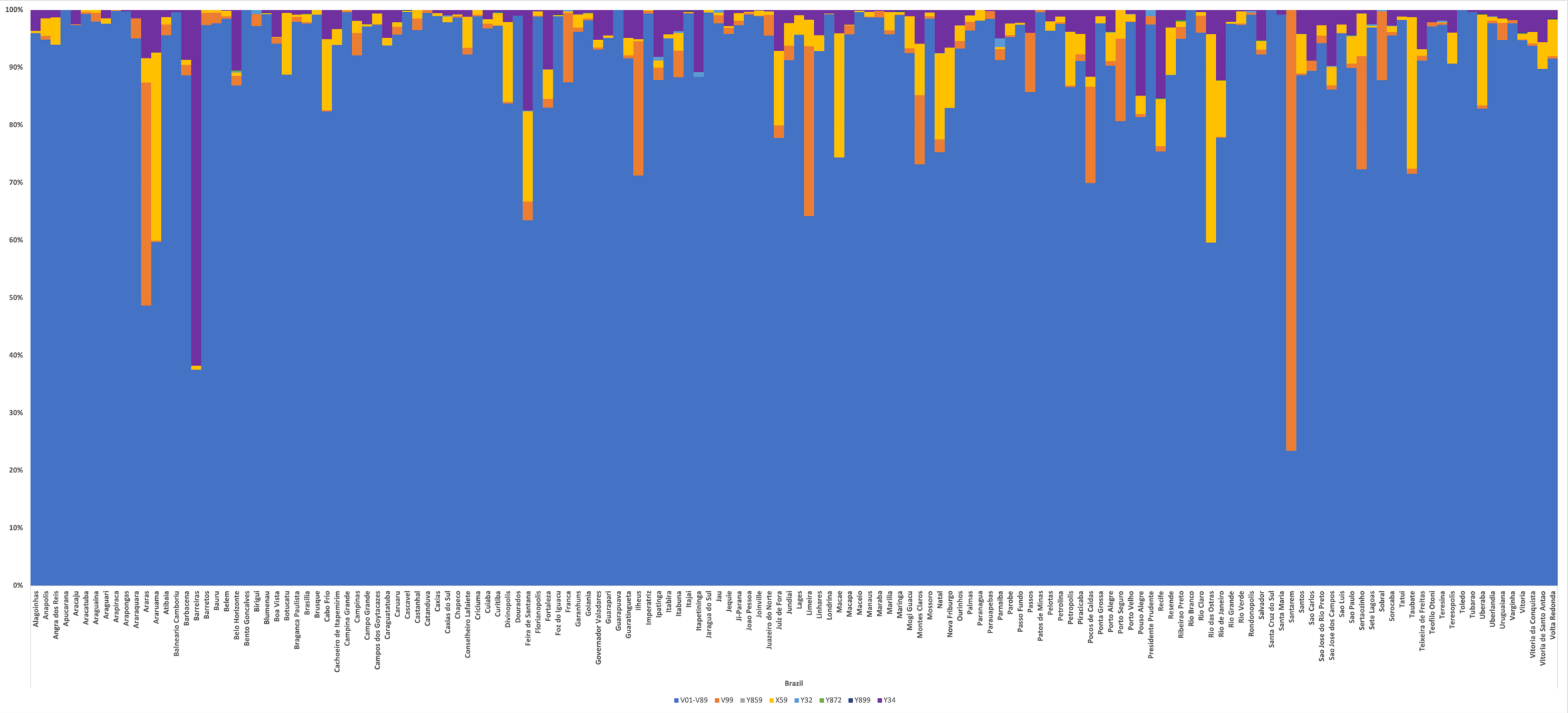
100%
90%
80%
70%
60%
50%
40%
30%
20%
10%
0%
Brazil
V01-V89
V99
Y859
X59
Y32
Y872
Y899
Y34

C) Mexico, Costa Rica, El Salvador, Guatemala, and Panama

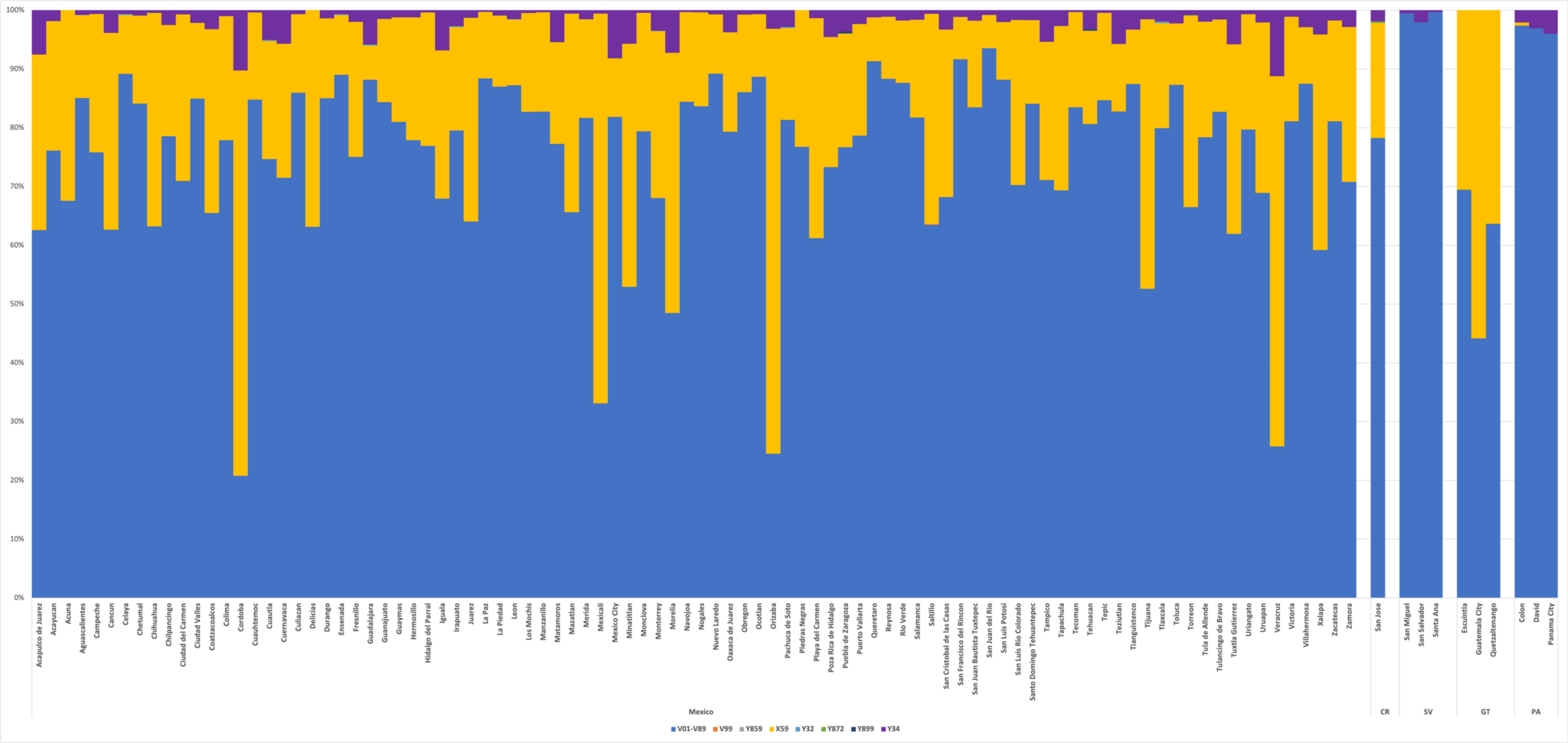

Supplementary Table 3a. Female 5-year road traffic mortality by age group by country, medians and interquartile ranges (IQRs) of cities.

Age Group (years)	Argentina	Brazil	Chile	Colombia	Costa Rica	El Salvador	Guatemala	Mexico	Panama	Peru
	N=33	N=152	N=21	N=35	N=1	N=3	N=3	N=92	N=3	N=23
	Median (IQR)	Median (IQR)	Median (IQR)	Median (IQR)	Median (IQR)	Median (IQR)	Median (IQR)	Median (IQR)	Median (IQR)	Median (IQR)
**<5**	2.6 (1.4, 3.6)	2.6 (1.2, 4.4)	3.4 (2.6, 5.8)	2.7 (1.2, 4.0)	2.6 (-)	1.9 (0.0, 7.8)	1.5 (0.9, 2.8)	5.1 (3.4, 7.0)	4.1 (1.6, 5.0)	5.6 (3.0, 10.8)
**5 to 9**	1.9 (1.0, 3.1)	2.2 (1.0, 3.9)	1.6 (0, 2.6)	1.4 (0, 3.3)	0.4 (-)	1.7 (1.1, 5.4)	0.9 (0, 3.8)	2.2 (1.3, 3.7)	2.2 (1.6, 5.2)	3.6 (1.7, 6.6)
**10 to 14**	2.0 (1.5, 2.9)	2.7 (1.6, 4.3)	2.7 (1.1, 4.9)	2.4 (1.0, 3.5)	.7 (-)	6.8 (1.5, 8.4)	0.8 (0.7, 1.6)	2.7 (1.7, 4.0)	2.2 (1.9, 5.5)	2.5 (1.1, 4.2)
**15 to 19**	6.7 (3.6, 8.0)	7.9 (5.6, 12.1)	5.3 (3.9, 8.1)	5.6 (3.7, 9.2)	3.1 (-)	3.3 (2.2, 6.3)	2.8 (2.1, 3.4)	7.5 (4.7, 9.3)	4.4 (3.4, 7.8)	3.7 (1.8, 8.3)
**20 to 24**	6.9 (5.4, 10.2)	10.6 (7.2, 14.7)	7.2 (4.6, 9.6)	6.7 (4.5, 10.6)	5.9 (-)	4.9 (4.7, 9.8)	3.4 (0, 11.3)	8.3 (6.3, 11.3)	6.9 (5.2, 12.2)	6.3 (2.8, 9.1)
**25 to 29**	6.9 (5.2, 9.3)	8.8 (6.5, 11.4)	4.9 (3.7, 6.9)	7.0 (3.6, 10.2)	3.5 (-)	3.6 (3.0, 15.6)	4.7 (3.1, 10.5)	7.8 (5.3, 10.3)	5.4 (5.0, 6.5)	7.1 (3.7, 10.1)
**30 to 34**	6.4 (4.0, 9.3)	8.3 (5.9, 11.8)	4.9 (3.0, 5.7)	5.6 (3.5, 9.8)	4.4 (-)	5.9 (4.8, 10.3)	4.3 (3.5, 4.9)	7.0 (5.7, 9.2)	2.6 (0, 3.8)	7.4 (5.7, 11.9)
**35 to 29**	5.1 (3.3, 6.5)	7.8 (5.5, 11.3)	5.5 (3.8, 7.6)	6.2 (4.8, 10.2)	3.4 (-)	6.1 (5.7, 9.8)	8.8 (5.0, 9.2)	7.8 (5.3, 11.0)	5.1 (3.5, 5.5)	6.8 (4.2, 12.3)
**40 to 44**	6.5 (3.7, 7.7)	9.0 (6.1, 11.9)	5.1 (2.9, 5.7)	6.5 (5.0, 9.6)	5.1 (-)	2.3 (0, 4.9)	4.6 (4.5, 7.2)	7.1 (5.2, 9.6)	5.4 (4.8, 11.5)	8.1 (4.5, 12.8)
**45 to 49**	5.0 (3.9, 7.8)	8.3 (5.3, 13.1)	7.0 (4.7, 7.7)	5.4 (3.6, 7.2)	5.2 (-)	5.2 (3.3, 7.7)	4.5 (3.9, 16.1)	7.9 (5.4, 10.7)	3.1 (2.8, 3.1)	10.0 (5.1, 15.5)
**50 to 54**	5.8 (3.5, 9.2)	9.2 (6.7, 13.6)	6.4 (4.6, 10.8)	4.9 (3.6, 8.0)	2.5 (-)	4.1 (3.1, 6.6)	5.6 (4.2, 6.6)	9.5 (7.2, 11.5)	5.5 (0, 11.0)	11.7 (5.5, 23.3)
**55 to 59**	7.9 (4.4, 10.9)	9.4 (5.7, 13.0)	6.4 (5.4, 10.4)	8.3 (6.0, 11.8)	6.0 (-)	9.7 (9.4, 14.7)	7.9 (0, 19.9)	10.1 (5.6, 14.6)	5.6 (3.8, 14.0)	17.7 (10.2, 27.2)
**60 to 64**	8.8 (5.1, 12.5)	11.5 (7.4, 15.6)	9.3 (6.7, 14.3)	9.5 (7.5, 12.5)	8.1 (-)	13.0 (12.5, 16.5)	13.8 (11.3, 16.3)	14.4 (10.0, 21.0)	9.8 (0, 30.0)	16.2 (9.7, 27.9)
**65 to 69**	7.0 (3.8, 11.3)	13.8 (8.3, 18.8)	10.2 (4.5, 14.8)	12.0 (7.1, 19.4)	9.6 (-)	19.8 (0.0, 36.4)	11.9 (7.2, 21.0)	18.4 (10.1, 23.3)	15.5 (0, 18.7)	20.9 (14.3, 38.3)
**70 to 74**	12.2 (5.3, 16.6)	16.4 (10.4, 24.6)	11.1 (8.9, 17.5)	20.8 (16.0, 36.3)	23.0 (-)	20.3 (0.0, 32.1)	15.2 (0, 50.3)	21.6 (15.3, 29.7)	12.1 (9.0, 16.3)	32.8 (19.7, 44.4)
**75 to 79**	10.9 (3.9, 16.3)	20.5 (12.8, 31.1)	14.7 (10.4, 22.8)	27.3 (18.4, 39.5)	29.2 (-)	31.6 (10.4, 42.1)	25.5 (17.6, 44.7)	36.7 (26.6, 53.9)	12.7 (0, 21.5)	46.8 (26.6, 85.6)
**≥80**	22.5 (17.9, 37.4)	26.8 (17.7, 39.9)	16.0 (8.9, 24.0)	31.5 (23.1, 43.7)	97.1 (-)	33.4 (33.2, 76.3)	66.1 (45.3, 78.9)	91.6 (73.7, 117)	6.3 (0, 14.8)	152 (103, 231)

Supplementary Table 3b. Male 5-year road traffic mortality by age group by country, medians and interquartile ranges (IQRs) of cities.

Age Group (years)	Argentina N=33 Median (IQR)	Brazil N=152 Median (IQR)	Chile N=21 Median (IQR)	Colombia N=35 Median (IQR)	Costa Rica N=1 Median (IQR)	El Salvador N=3 Median (IQR)	Guatemala N=3 Median (IQR)	Mexico N=92 Median (IQR)	Panama N=3 Median (IQR)	Peru N=23 Median (IQR)
**<5**	4.0 (1.4, 6.2)	3.6 (2.4, 5.8)	3.8 (2.5, 5.6)	2.5 (1.3, 4.7)	1.5 (-)	3.7 (1.7, 5.1)	2.3 (2.1, 5.8)	6.4 (4.5, 9.8)	4.7 (3.8, 7.8)	6.1 (3.4, 15.4)
**5 to 9**	2.3 (1.5, 3.6)	3.9 (2.3, 5.8)	3.2 (2.0, 4.6)	3.2 (1.8, 5.2)	1.6 (-)	5.1 (3.6, 6.4)	1.4 (0.8, 4.6)	3.9 (2.3, 5.5)	2.6 (0, 3.3)	4.6 (2.4, 6.2)
**10 to 14**	3.8 (2.7, 5.0)	5.1 (3.5, 7.3)	2.2 (1.5, 3.0)	3.6 (2.4, 5.6)	2.6 (-)	12.4 (3.2, 12.5)	2.2 (1.7, 3.2)	5.7 (3.9, 8.4)	3.2 (0, 6.6)	4.3 (1.9, 6.1)
**15 to 19**	24.6 (19.0, 31.5)	31.1 (23.0, 39.6)	13.4 (7.3, 15.3)	23.3 (14.8, 30.0)	11.4 (-)	9.4 (9.0, 17.4)	14.7 (12.6, 26.9)	26.1 (18.7, 35.5)	15.6 (10.8, 17.5)	11.5 (7.5, 17.8)
**20 to 24**	41.0 (31.5, 46.3)	54.7 (43.1, 70.3)	22.8 (17.5, 31.5)	44.6 (39.2, 51.8)	28.9 (-)	19.0 (18.4, 24.5)	33.0 (19.7, 64.8)	41.4 (32.3, 53.4)	29.3 (25.4, 43.6)	26.8 (18.6, 40.9)
**25 to 29**	38.2 (30.2, 44.8)	49.2 (39.9, 64.3)	23.8 (19.8, 31.1)	40.8 (33.9, 58.1)	24.4 (-)	31.3 (26.2, 39.5)	38.2 (28.1, 66.2)	41.2 (32.3, 51.2)	33.2 (22.8, 48.0)	30.3 (21.4, 37.1)
**30 to 34**	30.2 (23.1, 42.1)	47.6 (37.8, 61.1)	22.9 (18.3, 27.8)	38.1 (31.2, 55.3)	21.7 (-)	38.6 (34.0, 53.8)	32.2 (24.4, 68.3)	40.3 (33.4, 52.9)	37.8 (21.4, 45.5)	26.2 (17.6, 37.0)
**35 to 29**	30.0 (23.5, 34.6)	44.6 (37.1, 59.3)	22.1 (15.8, 30.4)	36.1 (25.1, 48.8)	22.0 (-)	49.7 (40.4, 50.8)	46.1 (19.5, 99.9)	41.5 (31.9, 50.4)	30.7 (17.7, 38.5)	39.5 (25.7, 41.9)
**40 to 44**	26.5 (17.1, 30.5)	47.3 (36.8, 61.2)	27.4 (22.9, 34.9)	33.6 (23.4, 47.1)	23.4 (-)	39.4 (38.9, 47.9)	47.1 (15.8, 69.6)	38.2 (31.0, 48.4)	20.2 (16.1, 27.5)	34.1 (24.1, 53.9)
**45 to 49**	27.2 (21.4, 36.0)	48.9 (38.9, 62.7)	27.0 (19.2, 35.8)	36.6 (24.2, 46.9)	22.3 (-)	56.9 (40.8, 67.8)	36.6 (24.7, 44.6)	39.3 (27.7, 51.4)	25.7 (23.6, 46.5)	47.5 (33.1, 51.0)
**50 to 54**	27.9 (21.1, 35.8)	45.9 (37.6, 61.7)	34.9 (25.5, 40.2)	36.5 (26.2, 49.1)	23.8 (-)	42.3 (34.8, 53.8)	58.8 (29.1, 95.8)	44.3 (34.4, 52.0)	45.4 (23.2, 48.7)	43.0 (22.5, 51.8)
**55 to 59**	29.1 (23.7, 35.8)	49.4 (37.5, 67.4)	29.2 (25.1, 42.6)	36.5 (29.0, 58.2)	28.1 (-)	43.7 (30.4, 113)	71.9 (25.2, 110)	45.7 (39.4, 61.3)	29.3 (24.1, 45.1)	43.4 (34.5, 58.4)
**60 to 64**	34.7 (25.0, 41.1)	50.0 (37.0, 66.8)	41.7 (31.8, 47.6)	35.4 (29.5, 52.1)	31.2 (-)	56.8 (50.9, 74.1)	59.0 (26.6, 59.0)	50.0 (41.5, 63.8)	38.2 (23.9, 51.6)	56.3 (40.0, 83.1)
**65 to 69**	30.8 (21.6, 46.8)	50.2 (37.9, 65.6)	40.8 (31.6, 57.1)	51.5 (35.5, 64.1)	27.5 (-)	65.0 (60.6, 124)	85.7 (38.2, 90.4)	59.3 (45.0, 78.2)	35.0 (9.2, 59.8)	67.7 (45.5, 94.6)
**70 to 74**	34.9 (25.1, 47.8)	54.9 (39.6, 75.2)	48.8 (36.7, 57.7)	73.8 (53.2, 89.4)	48.1 (-)	91.5 (86.6, 95.9)	40.1 (38.7, 75.8)	68.9 (55.3, 94.9)	53.3 (41.4, 70.4)	86.3 (51.8, 131.8)
**75 to 79**	41.2 (29.1, 52.0)	67.4 (47.1, 91.7)	59.8 (45.4, 75.7)	92.3 (64.2, 141)	65.0 (-)	70.5 (67.5, 81.5)	77.8 (60.5, 186.3)	88.0 (69.6, 130)	56.3 (55.2, 60.9)	107 (66.3, 208)
**≥80**	48.3 (33.2, 70.9)	77.1 (59.0, 109)	65.4 (54.1, 74.4)	124 (96.5, 171)	146.8 (-)	146 (94.2, 267)	95.1 (71.1, 185)	161 (117, 198)	36.9 (31.5, 74.2)	233 (166, 415)

Supplementary Table 4. Pearson correlation matrix of exposures and covariates.

	Population Density in Built Up Area in 2010	Population Growth 2010-2016	Social Environment Index	GDP Per Capita in 2010	Patch Density (Fragmentation)	Area-Weighted Mean Nearest Neighbor (Islolation)	% City Built-Up	Intersection Density	Street Length Average	Streets per Node Average
**Population Growth 2010-2016***	-0.2055	1								
**Social Environment Index**	-0.1128	-0.1113	1							
**GDP Per Capita in 2010**	-0.2373	0.1543	0.2604	1						
**Patch Density (Fragmentation)**	-0.0353	0.0471	0.0588	-0.0395	1					
**Area-Weighted Mean Nearest Neighbor (Islolation)**	0.0246	-0.0206	-0.2482	0.0036	-0.3889	1				
**% City Built-Up**	0.0593	-0.0101	0.1695	0.0054	0.7422	-0.4176	1			
**Intersection Density**	0.1757	-0.0341	0.1973	-0.0102	0.6465	-0.4133	**0.9314**	1		
**Street Length Average**	-0.2034	0.0404	-0.134	0.1554	-0.2519	0.4339	-0.376	-0.4371	1	
**Streets per Node Average**	-0.2334	-0.0127	0.0765	0.0259	-0.4046	0.0735	-0.1762	-0.1287	0.0204	1
**Urban Travel Delay Index**	0.5264	0.0633	0.1434	-0.0875	0.1711	-0.1859	0.3169	0.3556	-0.3031	-0.2136

Red = Negative correlation
Yellow = Correlation near 0
Green = Positive correlation

Supplementary Table 5. Sensitivity analysis results. A) In Complete Case analysis, only deaths originally coded as road traffic deaths are included (ICD10 V01-V89 with exceptions noted in Appendix II). B) Analyses included only cities with <20% road traffic deaths originating from ill-defined coded deaths. C) The proportion of a city's road traffic deaths originating from ill-defined coded deaths is included as a covariate in all models. Compare to estimates in Table 3 in the main manuscript.
RR were estimated using mixed effects generalized linear model regression with negative binomial distribution and robust standard errors. All RRs and 95% CIs reflect a difference of 1 standard deviation (SD) except as noted. All models are adjusted for a fixed effect for country, sex and 5-year age group. Model 1 is a single exposure model of each exposure and covariate. Model 2 includes all exposures and covariates in a multivariable model. Model 3 removes patch density and percent built up area from the model. Bolded values are statistically significant at P<0.05.

	**A) Complete Case**			**B) Cities <20% Ill-defined**			**C) Add Ill-defined % as Covariate**		
Measure	Model 1	Model 2	Model 3	Model 1	Model 2	Model 3	Model 1	Model 2	Model 3
**Population Measures**									
Population Density in Built Up Area in 2010	**0.87 (0.82, 0.92)**	**0.92 (0.86, 0.99)**	**0.92 (0.86, 0.98)**	**0.92 (0.87, 0.96)**	0.97 (0.92, 1.02)	0.97 (0.92, 1.01)	**0.90 (0.86, 0.94)**	**0.93 (0.89, 0.98)**	**0.94 (0.90, 0.98)**
Population Growth 2010-2016*	1.04 (0.99, 1.08)	1.03 (0.99, 1.08)	1.03 (0.99, 1.07)	**1.06 (1.02, 1.10)**	1.04 (1.00, 1.07)	1.04 (1.00, 1.07)	1.03 (0.99, 1.07)	1.02 (1.00, 1.06)	1.03 (1.00, 1.06)
**Social Environment Index**	**0.92 (0.85, 0.99)**	1.00 (0.92, 1.09)	1.00 (0.92, 1.08)	**0.78 (0.72, 0.84)**	**0.83 (0.75, 0.92)**	**0.83 (0.75, 0.92)**	**0.90 (0.85, 0.94)**	0.96 (0.91, 1.02)	0.97 (0.92, 1.02)
**GDP Per Capita in 2010**	**0.96 (0.93, 1.00)**	**0.96 (0.92, 0.99)**	**0.96 (0.93, 0.99)**	**0.94 (0.89, 1.00)**	0.99 (0.94, 1.04)	0.99 (0.94, 1.04)	**0.96 (0.93, 0.99)**	**0.96 (0.94, 0.98)**	**0.96 (0.94, 0.98)**
**Urban Landuse Measures**									
Patch Density (Fragmentation)	1.02 (0.97, 1.08)	0.99 (0.94, 1.05)		**0.90 (0.86, 0.93)**	0.99 (0.95, 1.04)		**0.91 (0.88, 0.94)**	0.99 (0.95, 1.03)	
Area-Weighted Mean Nearest Neighbor (Isolation)	**1.08 (1.02, 1.15)**	1.04 (0.99, 1.09)	1.04 (0.99, 1.09)	**1.08 (1.01, 1.14)**	0.99 (0.94, 1.03)	0.99 (0.95, 1.03)	**1.08 (1.05, 1.12)**	**1.05 (1.01, 1.08)**	**1.05 (1.01, 1.09)**
% City Built-Up	**0.86 (0.82, 0.91)**	1.02 (0.90, 1.17)		**0.87 (0.85, 0.90)**	1.02 (0.91, 1.13)		**0.89 (0.86, 0.91)**	0.99 (0.91, 1.07)	
**Road Network Measures**									
Intersection Density	**0.87 (0.83, 0.9)**	0.89 (0.78, 1.02)	**0.91 (0.86, 0.96)**	**0.88 (0.85, 0.90)**	0.90 (0.81, 1.01)	**0.92 (0.88, 0.95)**	**0.88 (0.86, 0.91)**	0.94 (0.87, 1.02)	**0.92 (0.89, 0.95)**
Street Length Average	1.05 (0.98, 1.13)	0.97 (0.89, 1.05)	0.97 (0.9, 1.05)	1.05 (0.98, 1.14)	0.99 (0.93, 1.05)	0.99 (0.93, 1.05)	1.03 (1.00, 1.07)	0.96 (0.92, 1.00)	0.96 (0.92, 1.00)
Streets per Node Average	**1.06 (1.02, 1.1)**	1.02 (0.97, 1.07)	1.02 (0.98, 1.06)	**1.06 (1.02, 1.10)**	1.03 (1.00, 1.07)	**1.03 (1.00, 1.07)**	**1.06 (1.02, 1.09)**	1.02 (0.99, 1.05)	1.02 (0.99, 1.05)
**Transportation Measures**									
Urban Travel Delay Index	**0.86 (0.82, 0.91)**	0.95 (0.89, 1.01)	0.95 (0.89, 1.01)	**0.90 (0.85, 0.94)**	0.98 (0.93, 1.04)	0.98 (0.93, 1.04)	**0.89 (0.86, 0.92)**	0.98 (0.94, 1.02)	0.98 (0.94, 1.02)
Presence of BRT or subway (vs none)**	**0.75 (0.68, 0.83)**	0.94 (0.86, 1.04)	0.95 (0.87, 1.04)	**0.76 (0.70, 0.83)**	0.93 (0.86, 1.01)	0.94 (0.87, 1.01)	**0.77 (0.71, 0.82)**	**0.93 (0.86, 0.99)**	**0.93 (0.87, 0.99)**

Appendix II: Description of specific ICD-10 codes used to identity road traffic deaths

We considered all deaths coded with codes V01-V89 to be road traffic deaths with the exception of these codes:

V81.2 – Occupant of railway train or railway vehicle injured in collision with or hit by rolling stock

V81.3 – Occupant of railway train or railway vehicle injured in collision with other object

V81.4 – Person injured while boarding or alighting from railway train or railway vehicle

V81.5 – Occupant of railway train or railway vehicle injured by fall in railway train or railway vehicle

V81.6 – Occupant of railway train or railway vehicle injured by fall from railway train or railway vehicle

V81.7 – Occupant of railway train or railway vehicle injured in derailment without antecedent collision

V81.8 – Occupant of railway train or railway vehicle injured in other specified railway accidents

V81.9 – Occupant of railway train or railway vehicle injured in unspecified railway accident

We included traffic and non-traffic collisions as described in the manuscript.

Y850 Sequelae of Motor-Vehicle Accidents deaths were also counted as road traffic deaths.

Appendix II: Detailed descriptions of exposure measures and their interpretation

Population Density in Built-up Areas. City population per square kilometers in all the urban patches inside the city geographic boundary as of 2010 or the closest year to 2010 for which official geographic boundaries were available. The population for each city was based on the projected population of all administrative units that comprise the city from official country estimates. Population is adjusted for United Nations' country-level population projections. Urban patch definition is described below in Patch Density variable.

Population Growth. Percent change in projected population in a city between the first and last year of the years of mortality data covered in the study (Supplementary Table 1).

Social Environment Index. This variable is the standardized mean of four values: proportion of a city's dwellings with a water connection inside the dwelling, proportion of the city's dwellings with a sewage connection, proportion of the city's households with overcrowding defined as 3 or more people per room, and proportion of the city's population 25 years or older with primary education or higher. These measures are based on the most recent year of census data for the country: AR 2010, BR 2010, CL 2002, CO 2007, CR 2011, SV 2007, GT 2002, MX 2010, PA 2010, and PE 2007.

Gross Domestic Product per Capita. City-level GDP in 2010 in 2011 international US Dollars created by Genaioli et al in 2013 and converted into gridded estimates by Kummu et al in 2015. GDP for each year 1990-2015 was estimated by these researchers by modeling data from government, survey and industry. Gridded estimates were matched to SALURBAL cities and GDP was extracted directly from matching administrative units or using population-weighted averages if a city boundaries crossed multiple administrative areas.

Gennaioli N, La Porta R, Lopez-de-Silanes F, Shleifer A, Human Capital and Regional Development, The Quarterly Journal of Economics, Volume 128, Issue 1, February 2013, Pages 105–164, https://doi.org/10.1093/qje/qjs050.

Kummu M, Taka M, & Guillaume J. Gridded global datasets for Gross Domestic Product and Human Development Index over 1990–2015. Sci Data 5, 180004 (2018). https://doi.org/10.1038/sdata.2018.4.

Patch Density. The number of urban patches divided by the total area of a city (in 100 hectares) where an urban patch is a contiguous urban development. To identify urban patches, 30 X 30m gridcells from the Global Urban Footprint (GUF) project from 2012 were used. For each built-up gridcell within the defined city boundaries, an algorithm classified them as urban if >50% of all gridcells within a one-km^2 area surrounding it were built-up cells, suburban if 25-50% of gridcells were built-up and rural if <25% of gridcells were built-up. Urban clusters were then created based on contiguous urban, suburban and urbanized open space gridcells (e.g., a park surrounded entirely by built-up area). Urban clusters were then merged if adjacent clusters had contiguous or intersecting buffer areas 25% larger than the clusters. The resulting agglomerations once no more merges were possible are the urban patches. The program FRAGSTATS 4.2 was used to calculate number of urban patches per city.

Area-Weighted Mean Nearest Neighbor. Mean distance (in meters) to the nearest urban patch within the geographic boundary of the city. This value is weighted by the area of each patch.

Percent Built-Up. Total urban area (sum of all urban patches in the city's geographic boundaries) divided by the total area a city and multiplied by 100. Ranges from 0 to 100.

Intersection Density. The number of intersections (a point where more than 2 streets meet) per area of a city in square kilometers. Intersections were extracted from street network OpenStreetMap data and included any intersections with >2 connected streets (i.e., cul-de-sacs and road bends represented as a node were excluded). Intersections per hectare.

Street Length Average. The average length of street segments in meters (a section of a street between two intersections) in a city. These data were extracted from OpenStreetMap data.

Street Node Average. The average number of streets that meet at intersections in a city. These data were extracted from OpenStreetMap data.

Urban Travel Delay Index. Calculated as the average increase in travel times in a city imposed by congestion for 30 randomly selected pairs of points in the city and measured during seven points in time during peak traffic hours of a typical weekday ($\frac{congested\ travel\ time - uncongested\ travel\ time}{uncongested\ travel\ time}$) using the Google Maps API.

BRT or Subway Presence. The presence or absence of a bus rapid transit (BRT) or subway system in a city as of 2017 using BRTData (https://brtdata.org).

APPENDIX IV: City-Level Age-Adjusted Road-Traffic Mortality Rate per 100,000 Population, 2010-2016

Lowest to highest rates per country. Capital city for each country indicated in parentheses. El Salvador and Panama rates do not include the full 2010-2016 period, see below.

Argentina

Urban Area Name	Age-Adjusted Road Traffic Mortality per 100,000 Population, 2010-2016
San Carlos de Bariloche	9.73
Buenos Aires (Capital)	9.76
Cordoba	9.83
Corrientes	12.07
Salta	12.57
Comodoro Rivadavia	12.69
Mar del Plata	13.14
Parana	13.17
Bahia Blanca	13.30
Concordia	13.31
Miguel de Tucuman-Tafi Viejo	13.84
Catamarca	14.08
San Luis	14.67
Santa Rosa	14.77
Mendoza	14.85
Tandil	14.92
Rosario	15.25
La Rioja	15.31
Rawson-Trelew	15.55
Neuquen-Plottier-Cipolletti	15.83
Santa Fe	16.75
Posadas	16.94
Villa Mercedes	17.30
Rio Cuarto	17.32
Resistencia	17.74
San Juan	17.76
Formosa	17.84
Zarate-Campana	18.15
Rio Gallegos	18.91
San Rafael	19.30
San Nicolas de los Arroyos	19.47
Jujuy	19.47
Santiago del Estero	21.01

Brazil

Urban Area Name	Age-Adjusted Road Traffic Mortality per 100,000 Population, 2010-2016	Urban Area Name	Age-Adjusted Road Traffic Mortality per 100,000 Population, 2010-2016
Salvador	11.64	Pelotas	18.03
Sao Paulo	12.72	Curitiba	18.11
Natal	13.71	Maceio	18.23
Porto Alegre	14.20	Conselheiro Lafaiete	18.27
Belem	14.54	Ourinhos	18.35
Presidente Prudente	15.01	Rio Claro	18.59
Caxias do Sul	15.10	Bento Goncalves	18.64
Barreiras	15.28	Pouso Alegre	18.80
Uruguaiana	15.32	Sao Carlos	18.90
Passos	15.40	Vitoria	18.91
Taubate	15.53	Pocos de Caldas	18.96
Sao Luis	15.71	Sertaozinho	18.97
Bauru	15.95	Florianopolis	18.98
Rio de Janeiro	16.04	Rio Grande	19.07
Juiz de Fora	16.25	Aracaju	19.12
Campinas	16.25	Guaratingueta	19.15
Marilia	16.33	Botucatu	19.18
Franca	16.48	Porto Seguro	19.20
Birigui	16.64	Ipatinga	19.29
Sao Jose dos Campos	16.67	Feira de Santana	19.50
Itabira	16.73	Criciuma	19.70
Jundiai	16.77	Brasilia (Capital)	20.22
Santa Cruz do Sul	16.86	Araras	20.23
Volta Redonda	16.98	Mogi Guacu	20.23
Recife	17.18	Aracatuba	20.38
Joao Pessoa	17.39	Braganca Paulista	20.74
Petropolis	17.43	Jau	20.82
Manaus	17.43	Macapa	20.93
Santa Maria	17.50	Tatui	20.95
Santos	17.54	Balneario Camboriu	21.05
Belo Horizonte	17.56	Sao Jose do Rio Preto	21.06
Sorocaba	17.63	Barretos	21.09
Piracicaba	17.80	Itabuna	21.14
Limeira	17.85	Nova Friburgo	21.29
Araraquara	17.89	Itapetininga	21.45
Resende	17.94	Rio Branco	21.48
Ribeirao Preto	17.95	Sete Lagoas	21.49
Varginha	18.02	Catanduva	21.60

Urban Area Name	Age-Adjusted Road Traffic Mortality per 100,000 Population, 2010-2016
Passo Fundo	21.62
Cabo Frio	21.69
Uberaba	21.85
Barbacena	22.00
Caruaru	22.04
Joinville	22.11
Vitoria de Santo Antao	22.12
Divinopolis	22.41
Fortaleza	22.46
Ilheus	22.76
Uberlandia	22.81
Mossoro	23.24
Caraguatatuba	23.28
Angra dos Reis	23.72
Montes Claros	23.75
Patos de Minas	23.81
Ponta Grossa	23.83
Parobe	24.13
Rio das Ostras	24.16
Campo Grande	24.17
Araguari	24.21
Alagoinhas	24.28
Brusque	24.43
Campina Grande	25.12
Macae	25.65
Londrina	25.69
Goiania	25.94
Araruama	26.10
Teresopolis	26.32
Chapeco	26.34
Paranagua	26.46
Governador Valadares	26.75
Teofilo Otoni	27.60
Atibaia	27.62
Lages	28.03
Jaragua do Sul	28.15
Santarem	28.17
Guarapari	28.24
Maringa	28.31
Garanhuns	28.50

Urban Area Name	Age-Adjusted Road Traffic Mortality per 100,000 Population, 2010-2016
Tubarao	28.53
Cuiaba	28.69
Jequie	28.77
Itajai	28.88
Blumenau	28.94
Apucarana	29.14
Teresina	29.18
Juazeiro do Norte	29.27
Petrolina	29.39
Parnaiba	30.20
Cascavel	30.36
Foz do Iguacu	30.50
Vitoria da Conquista	31.54
Rio Verde	31.71
Castanhal	32.32
Palmas	32.33
Campos dos Goytacazes	32.44
Guarapuava	33.41
Imperatriz	33.41
Toledo	33.61
Sobral	33.72
Linhares	33.72
Arapongas	33.85
Anapolis	34.40
Arapiraca	34.49
Dourados	34.53
Cachoeiro de Itapemirim	34.69
Araguaina	35.20
Teixeira de Freitas	35.22
Boa Vista	36.20
Porto Velho	37.85
Maraba	41.36
Rondonopolis	41.67
Parauapebas	43.78
Ji-Parana	46.41
Caxias	48.24

Chile

Urban Area Name	Age-Adjusted Road Traffic Mortality per 100,000 Population, 2010-2016
Valparaiso-Vina del Mar	7.56
Santiago (Capital)	9.18
Valdivia	10.34
Quillota	10.86
Concepcion	11.58
La Serena-Coquimbo	11.78
Osorno	12.69
Talca	13.18
Temuco	13.51
Chillan	13.60
San Antonio	13.67
Punta Arenas	13.88
Antofagasta	14.36
Iquique	14.54
Curico	15.21
Rancagua	15.67
Copiapo	15.73
Puerto Montt	15.78
Los Angeles	18.58
Arica	20.90
Calama	23.77

Colombia

Urban Area Name	Age-Adjusted Road Traffic Mortality per 100,000 Population, 2010-2016
Barranquilla	8.85
Cartagena	10.81
Bogota (Capital)	11.42
Manizales	11.43
Monteria	12.10
Bucaramanga	13.82
Medellin	15.89
Armenia	15.92
Pasto	16.00
Fusagasuga	16.60
Ibague	16.85
Cucuta	16.90
Pereira	17.05
Girardot	17.57
Santa Marta	17.71
Duitama	17.74
Popayan	17.99
Sogamoso	18.07
Cali	18.46
Sincelejo	18.87
Tunja	19.64
Apartado	21.39
Palmira	22.22
Tulua	22.23
Quibdo	22.31
Cartago	22.82
Villavicencio	23.18
Valledupar	23.53
Neiva	25.44
Florencia	25.53
Buga	26.92
Barrancabermeja	30.42
Buenaventura	30.89
Riohacha	38.98
Yopal	41.78

Costa Rica

Urban Area Name	Age-Adjusted Road Traffic Mortality per 100,000 Population, 2010-2016
San Jose (Capital)	13.48

El Salvador

Urban Area Name	Age-Adjusted Road Traffic Mortality per 100,000 Population, 2010-2014
San Salvador (Capital)	16.21
San Miguel	18.44
Santa Ana	24.48

Guatemala

Urban Area Name	Age-Adjusted Road Traffic Mortality per 100,000 Population, 2010-2016
Guatemala City (Capital)	11.90
Quetzaltenango	19.92
Escuintla	30.08

Mexico

Urban Area Name	Age-Adjusted Road Traffic Mortality per 100,000 Population, 2010-2016
Mexico City (Capital)	13.23
Teziutlan	13.46
Merida	13.65
Veracruz	13.80
Cuernavaca	13.93
Cordoba	13.95
Orizaba	14.00
Minatitlan	14.13
Cancun	14.25
Tuxtla Gutierrez	15.11
Xalapa	15.91
San Cristobal de las Casas	16.12
Tampico	16.21
Coatzacoalcos	16.49
Tianguistenco	16.75
Campeche	17.15
Toluca	17.33
Puebla de Zaragoza	17.59
Puerto Vallarta	17.62
Monterrey	18.05
Poza Rica de Hidalgo	18.50
Ciudad del Carmen	18.57
Cuautla	18.63
Guanajuato	18.68
Uriangato	18.69
Tehuacan	18.74
Piedras Negras	19.16
Saltillo	19.46
Playa del Carmen	19.79
Matamoros	19.79
Mexicali	19.83
Juarez	20.02
Guadalajara	20.11
Acuna	20.23
San Luis Potosi	20.29
La Paz	20.34
Leon	20.45
Pachuca de Soto	20.55
Acapulco de Juarez	20.93

Urban Area Name	Age-Adjusted Road Traffic Mortality per 100,000 Population, 2010-2016
Colima	20.99
Tula de Allende	21.13
Tlaxcala	21.25
Tulancingo de Bravo	21.25
Monclova	21.31
Acayucan	21.58
San Luis Rio Colorado	22.07
Tijuana	22.22
Ciudad Valles	22.55
Oaxaca de Juarez	22.61
Queretaro	22.65
Hermosillo	22.65
Tapachula	22.75
Reynosa	22.86
Victoria	22.86
Guaymas	23.06
Chetumal	23.13
Rio Verde	23.15
Chihuahua	23.51
Iguala	23.64
San Juan del Rio	23.75
Mazatlan	23.76
Aguascalientes	23.91
San Francisco del Rincon	23.97
San Juan Bautista Tuxtepec	24.22
Santo Domingo Tehuantepec	24.87
Morelia	25.04
Delicias	25.64
Obregon	25.88
Nuevo Laredo	25.90
Villahermosa	26.27
Nogales	26.36
Manzanillo	26.61
Ensenada	27.59
Zamora	27.80
Tepic	28.26
Celaya	28.34
Torreon	28.48
Uruapan	29.58
La Piedad	30.10

Urban Area Name	Age-Adjusted Road Traffic Mortality per 100,000 Population, 2010-2016
Navojoa	30.56
Los Mochis	31.57
Chilpancingo	31.60
Ocotlan	31.61
Durango	32.06
Tecoman	32.53
Zacatecas	33.32
Irapuato	33.74
Fresnillo	33.96
Salamanca	34.14
Culiacan	35.14
Hidalgo del Parral	38.19
Cuauhtemoc	48.94

Panama

Urban Area Name	Age-Adjusted Road Traffic Mortality per 100,000 Population, 2012-2016
Panama City (Capital)	11.27
David	15.34
Colon	17.79

Peru

Urban Area Name	Age-Adjusted Road Traffic Mortality per 100,000 Population, 2010-2016
Piura	8.58
Lima (Capital)	11.24
Trujillo	15.00
Chiclayo	16.21
Iquitos	18.03
Ica	18.29
Huaraz	18.41
Cajamarca	18.50
Chincha Alta	19.26
Pucallpa	20.83
Sullana	21.96
Arequipa	22.15
Pisco	23.64
Chimbote	23.80
Tumbes	25.49
Huancayo	26.68
Tacna	27.22
Huanuco	27.43
Tarapoto	27.65
Ayacucho	29.16
Cusco	37.36
Puno	46.50
Juliaca	66.58
